# Hepatoma Derived Growth Factor Enhances Oligodendrocyte Genesis from Subventricular Zone Precursor Cells

**DOI:** 10.1177/17590914221086340

**Published:** 2022-03-16

**Authors:** Yutong Li, Nicole Leanne Dittmann, Adrianne Eve, Scovil Watson, Monique Marylin Alves de Almeida, Tim Footz, Anastassia Voronova

**Affiliations:** 1 Department of Medical Genetics, Faculty of Medicine and Dentistry, University of Alberta, Edmonton, Alberta, T6G 2H7, Canada; 2 Neuroscience and Mental Health Institute, Faculty of Medicine and Dentistry, University of Alberta, Edmonton, Alberta, T6G 2E1, Canada; 3 Women and Children’s Health Research Institute, 5-083 Edmonton Clinic Health Academy, University of Alberta, 11405 87 Avenue NW Edmonton, Alberta, Canada, T6G 1C9; 4 Department of Cell Biology, Faculty of Medicine and Dentistry, University of Alberta, Edmonton, Alberta, T6G 2H7, Canada; 5 Multiple Sclerosis Centre, Faculty of Medicine and Dentistry, University of Alberta, Edmonton, Alberta, T6G 2H7, Canada

**Keywords:** oligodendrocyte, SVZ, regeneration, neural stem cell, OPC, NCL

## Abstract

Oligodendrocytes, the myelinating cells of the central nervous system (CNS), perform vital functions in neural protection and communication, as well as cognition. Enhanced production of oligodendrocytes has been identified as a therapeutic approach for neurodegenerative and neurodevelopmental disorders. In the postnatal brain, oligodendrocytes are generated from the neural stem and precursor cells (NPCs) in the subventricular zone (SVZ) and parenchymal oligodendrocyte precursor cells (OPCs). Here, we demonstrate exogenous Hepatoma Derived Growth Factor (HDGF) enhances oligodendrocyte genesis from murine postnatal SVZ NPCs *in vitro* without affecting neurogenesis or astrogliogenesis. We further show that this is achieved by increasing proliferation of both NPCs and OPCs, as well as OPC differentiation into oligodendrocytes. *In vivo* results demonstrate that intracerebroventricular infusion of HDGF leads to increased oligodendrocyte genesis from SVZ NPCs, as well as OPC proliferation. Our results demonstrate a novel role for HDGF in regulating SVZ precursor cell proliferation and oligodendrocyte differentiation.

**Summary Statement:**

Hepatoma derived growth factor (HDGF) is produced by neurons. However, its role in the central nervous system is largely unknown. We demonstrate HDGF enhances i) oligodendrocyte formation from subventricular zone neural stem cells, and ii) oligodendrocyte precursor proliferation *in vitro* and *in vivo*.

## Introduction

Adult neural stem and precursor cells (NPCs) have the ability to self-renew and replace differentiated central nervous system (CNS) cells. The adult mammalian brain hosts two NPC niches: the subgranular zone in the hippocampus, and subventricular zone (SVZ) that lines the lateral ventricles (Chaker *et al*., 2016; Obernier and Alvarez-Buylla, 2019; Bacigaluppi *et al*., 2020). While subgranular zone NPCs normally give rise only to neurons and astrocytes, SVZ NPCs maintain their ability to differentiate into neurons, astrocytes and oligodendrocytes throughout life (Levison and Goldman, 1993; Steiner *et al*., 2004; Capilla-Gonzalez *et al*., 2013; Maki *et al*., 2013 and reviewed in Goldman, 1995; Obernier and Alvarez-Buylla, 2019). Thus, the SVZ NPCs remain multipotent, which makes them an interesting model for studying stem cell fate regulation.

For the purpose of this manuscript, we define “oligodendrocyte genesis” as formation of oligodendrocytes, where NPC to OPC (oligodendrocyte precursor cell) transition is termed as “commitment”, and OPC to oligodendrocyte transition is termed as “differentiation” (Butovsky *et al*., 2006; Gonzalez-Perez and Alvarez-Buylla, 2011; Voronova *et al*., 2017; Lakshman *et al*., 2021; Watson *et al*., 2021).

During early postnatal development, SVZ NPCs generate the majority of OPCs that populate the brain parenchyma, which then differentiate into oligodendrocytes or remain as slowly-dividing adult parenchymal OPCs (Polito and Reynolds, 2005; Kessaris *et al*., 2006; Kang *et al*., 2010). These parenchymal OPCs can be repopulated by the newly born SVZ OPCs (Cayre *et al*., 2006; Menn *et al*., 2006; Serwanski *et al*., 2018; Đặng *et al*., 2019). However, the contribution of adult SVZ NPCs to brain maintenance extends beyond OPC regeneration. *In vitro*, postnatal and adult dorsal SVZ NPCs preferentially give rise to oligodendroglial lineage cells, suggesting that SVZ NPCs generate oligodendrocytes in a region specific manner (Levison and Goldman, 1993; Ortega *et al*., 2013). This was recently confirmed with SVZ NPC lineage single-cell RNA-sequencing analysis (Cebrian Silla *et al*., 2021). When postnatal and adult SVZ NPCs are transplanted into the brains of shiverer mice that are devoid of myelin basic protein (MBP), they are able to migrate and form MBP^+^ oligodendrocytes, which myelinate the brain (Cayre *et al*., 2006). In the healthy adult brain, a subset of SVZ NPCs forms oligodendrocytes in the corpus callosum, striatum and fimbria fornix (Menn *et al*., 2006). Interestingly, while adult SVZ neurogenesis declines with age, adult SVZ oligodendrocyte genesis is stable throughout the aging process, which highlights SVZ NPC potential oligodendrogenic ability throughout life (Capilla-Gonzalez *et al*., 2013). Although parenchymal OPCs are often considered the “first line defenders” in a demyelination injury, SVZ NPCs are active contributors to oligodendrocyte genesis in the demyelinated CNS (central nervous system) in a region specific manner (Nait-Oumesmar *et al*., 1999; Picard-Riera *et al*., 2002; Menn *et al*., 2006; Aguirre and Gallo, 2007; Jablonska *et al*., 2010; Xing *et al*., 2014; Brousse *et al*., 2015; Delgado *et al*., 2021). Together, these studies suggest that SVZ NPCs are important contributors of oligodendrocyte genesis in a healthy and demyelinated CNS.

*De novo* oligodendrocyte genesis is important to study as stimulation of new oligodendrocyte formation and/or myelination promotes memory formation in aged mice, rescues aberrant social behavior in a mouse model of a neurodevelopmental disorder, and enhances regeneration in rodent models of neurodegenerative disorders (McKenzie *et al*., 2014; Barak *et al*., 2019; Steadman *et al*., 2019; Wang *et al*., 2020; Chen *et al*., 2021 and reviewed in Xin and Chan, 2020).

How can SVZ NPCs be recruited for enhanced oligodendrocyte genesis? It was recently shown that the transcriptome of activated SVZ NPCs closely resembles that of forebrain embryonic radial glial cells (neural stem cells) (Yuzwa *et al*., 2017; Borrett *et al*., 2020). In line with this, several molecules that were found to regulate developmental oligodendrocyte formation (Rowitch and Kriegstein, 2010), have been shown to regulate adult SVZ NPC fates (Xapelli *et al*., 2014). For example, epidermal growth factor (EGF) enhances oligodendrocyte formation from embryonic glial progenitors and postnatal or adult SVZ NPCs *in vitro* (Aguirre and Gallo, 2007; Gonzalez-Perez and Quiñones-Hinojosa, 2010; Yang *et al*., 2017a). Aguirre *et al*. elegantly demonstrated that overexpression of EGF receptor (EGFR) in the oligodendroglial lineage leads to increased oligodendrocyte generation and myelination in developing mice as well as in mice subjected to focal demyelination (Aguirre *et al*., 2007). In accordance, reduced EGFR signalling leads to a reduction in oligodendrocytes and myelination in the developing and demyelinated CNS at least in part via attenuation of SVZ oligodendroglial cell genesis (Aguirre *et al*., 2007; Aguirre and Gallo, 2007). Endothelin-1 (ET-1) regulates oligodendrocyte formation in the developing brain by promoting OPC proliferation and migration while restricting OPC differentiation (Gadea *et al*., 2009). In agreement, mice that lack ET-1 in Nestin^+^ postnatal NPCs have reduced SVZ OPCs in the developing postnatal brain, while brain slices cultured in the presence of ET-1 have an increased number of OPCs (Adams *et al*., 2020). Furthermore, mice with a conditional knockout of ET-1 in adult SVZ NPCs subjected to demyelination also have decreased SVZ OPC proliferation (Adams *et al*., 2020). Oligodendrocyte genesis from neural stem cells is also regulated by paracrine factors secreted from neighboring cells. For example, cortical inhibitory neurons secrete over 50 paracrine ligands that instruct embryonic radial glial cells to form oligodendrocytes (Voronova *et al*., 2017). One of these interneuron-secreted molecules is fractalkine (CX3CL1), which was shown to regulate developmental cortical oligodendrocyte genesis (Voronova *et al*., 2017). With regard to SVZ NPCs, fractalkine promotes postnatal oligodendrocyte genesis *in vitro* and adult oligodendrocyte genesis *in vivo* in a normal brain (Watson *et al*., 2021). While hepatoma derived growth factor (HDGF) is also expressed in cortical interneurons and has been predicted to regulate developmental oligodendrocyte formation (Voronova *et al*., 2017), its effect on NPCs is not known.

HDGF was first isolated from the Huh-7 hepatoma cell line (Wanschura *et al*., 1996) and is primarily known for its role in cancer cell proliferation (Bao *et al*., 2014). In the murine brain, HDGF is expressed from embryonic day (E) 13 until at least two years of age (Zhou *et al*., 2004). *Hdgf* mRNA is detected in hippocampal, cerebellar and cortical neurons *in vivo* and *in vitro* (Zhou *et al*., 2004; Voronova *et al*., 2017), and HDGF protein is secreted by hippocampal neurons and mouse neuroblastoma Neuro2a cells *in vitro* (Zhou *et al*., 2004). In addition to neurons, HDGF is also expressed in astrocytes, oligodendrocytes and microglia (Crossin *et al*., 1997; El-Tahir *et al*., 2006). Interestingly, HDGF knockout mice are viable and have no overt phenotype (Gallitzendoerfer *et al*., 2008). This indicates that endogenous HDGF is dispensable for normal development or that other HDGF family members, such as HDGF related proteins 1–4 and/or Lens epithelial derived growth factor, could compensate for a lack of HDGF (El-Tahir *et al*., 2006; Gallitzendoerfer *et al*., 2008). In a disease state, endogenous HDGF regulates glioma cell proliferation, migration and invasion (Song *et al*., 2014). Moreover, knockdown of endogenous HDGF enhances glioma cell sensitivity to temolozolomide, a brain tumour chemotherapy drug (Song *et al*., 2014). Exogenous HDGF has been proposed to act as a protective and potentially a pro-regenerative factor due to its anti-apoptotic and neurotrophic effects on neurons (Zhou *et al*., 2004). However, the effect of exogenous HDGF on other CNS cells is not known.

Here, we determined the effect of exogenous HDGF on SVZ NPCs and OPCs. Our results demonstrate that HDGF increases oligodendrocyte formation from murine postnatal SVZ NPCs in culture by enhancing SVZ NPC and OPC proliferation, as well as SVZ OPC differentiation. *In vivo* infusion of exogenous HDGF into the adult murine brain lateral ventricle increases oligodendrocyte genesis from SVZ NPCs, as well as OPC proliferation. Together, our results identify HDGF as a novel pro-oligodendrogenic molecule that can modulate SVZ precursor fate.

## Materials and Methods

### Mice

For primary cultures, timed pregnant wild-type CD1 mice were purchased from Charles River. CD1 pups at postnatal day (P) 7 were euthanized and their SVZ was microdissected. For *in vivo* experiments, 3 month old male and female NPC lineage tracing mice (NestinCre^ERT2^;RosaYFP^STOP/+^) were used. NestinCre^ERT2^ (C57BL/6-Tg(Nes-cre/ERT2) were obtained from (Imayoshi *et al*., 2006) and RosaYFP^STOP/STOP^ (B6.129X1-Gt(ROSA)26Sortm1(EYFP)Cos/J; RRID:IMSR_JAX:006148) mice were obtained from Jackson Laboratories. NestinCre^ERT2^ males and RosaYFP^STOP/STOP^ females were bred to create NestinCre^ERT2^;RosaYFP^STOP/+^ progeny, which were used for HDGF infusion experiments. Mice younger than 21 days of age were euthanized using CO_2_, and mice older than 21 days of age were euthanized with Euthansol (Western Drug Distribution Center Limited, WDDC) followed by intracardiac perfusion with Hanks Basic Saline Solution (HBSS, Invitrogen) and then 4% paraformaldehyde (PFA, Acros).

### HDGF Intracerebroventricular (ICV) Infusion

3-month old NestinCre^ERT2^;RosaYFP^STOP/+^ mice were injected for 5 days with 3 mg tamoxifen (Sigma) dissolved in 10% ethanol (Commercial Alcohols) and 90% sunflower seed oil (Sigma). 72 h after the last tamoxifen injection, mice were subjected to intracerebral ventricular (ICV) stereotaxic surgery. Mice were anaesthetized via isofluorane inhalation and placed in a stereotaxic frame. For the 1-time injection, 10 ng of HDGF (Abcam) or vehicle-control (0.2 mM TRIS pH 7.5, 0.01 mM EDTA, 0.01 mM DTT, 0.1% glycerol in 1xPhosphate Buffered Saline [PBS]) was injected into right ventricle of the brain at the rate of 0.1 μl/min. Overall, 1 μl was injected. For the 7-day infusion, osmotic minipumps (model 1007D, Alzet) were filled with 0.83 ng/ml HDGF diluted in 0.2% Bovine Serum Albumin (BSA) in 1x PBS, or vehicle-control (VC; 0.2 mM TRIS pH 7.5, 0.01 mM EDTA, 0.01 mM DTT, 0.1% glycerol) diluted in 0.2% BSA in 1xPBS. Minipumps were connected to cannulas that targeted the right ventricle. Overall, 12 μl or 10 ng of HDGF per day was delivered. The following coordinates were used for both infusion timelines: −1.000 medio-lateral, −0.300 anterior-posterior, −2.500 dorso-ventral. 24h before euthanasia, 100 mg/kg Bromodeoxyuridine (BrdU) (Sigma) was injected intraperitoneally.

### Primary Cultures

#### A) primary neurospheres:

Primary neurospheres were generated according to (Coles-Takabe *et al*., 2008; Storer *et al*., 2018). Briefly, P7 SVZ was microdissected and mechanically triturated in serum-free media (SFM: Dulbecco’s Modified Eagle Medium low glucose [DMEM, Gibco], Ham’s F-12 Nutrient Mixture [F12, Gibco], 0.6% glucose [Sigma], 0.1125% Sodium Bicarbonate [NaHCO3, Gibco], 5 mM N-2-hydroxyethylpiperazine-N-2-ethane sulfonic acid [HEPES, Gibco], 100 μg/mL L-glutamine [Lonza], 1% Penicillin-Streptomycin [Pen/Strep, Lonza]) supplemented with 2% B27 (Invitrogen), 10 ng/mL FGF (fibroblast growth factor, Peprotech), 20 ng/mL EGF (epidermal growth factor, Peprotech), and 2 μg/ml heparin sodium salt (Sigma) (herein referred to as neurosphere media). The cells were then centrifuged at 465g for 7 min, after which the supernatant was decanted and cells were resuspended in 1mL of neurosphere media. The cell suspension was filtered with a 40 μm strainer to obtain single cell suspension and get rid of any tissue debris. The cells were plated in neurosphere media at a clonal density of 10 cells/μL (Coles-Takabe *et al*., 2008; Storer *et al*., 2018).

#### B) secondary neurosphere cultures:

At 5-6 days *in vitro* (DIV), the floating primary neurospheres were collected and dissociated with 0.025% trypsin (Hyclone). After the cell pellet was washed twice in SFM, the cell solution was filtered through a 40 μm strainer to obtain a single cell suspension. Secondary neurospheres were generated by plating dissociated primary neurosphere cells at clonal density of 2 cells/μl in neurosphere media (Coles-Takabe *et al*., 2008; Storer *et al*., 2018) in the presence of 10 ng/ml HDGF or VC (0.2 mM TRIS pH 7.5, 0.01 mM EDTA, 0.01 mM DTT, 0.1% glycerol in 1xPBS) and cultured for 7 DIV.

#### C) NPC monolayer cultures:

Primary NPC monolayers were generated according to (Watson *et al*., 2021). Briefly, dissociated primary neurosphere cells were plated at 39,500 cells/cm^2^ on 12 mm glass coverslips (0.13-0.17 mm thickness, Fisher) coated with 40 μg/ml Poly-D-Lysine (Sigma) and 4 μg/ml laminin (Corning). Cells were cultured in SFM supplemented with 2% B27, 10 ng/ml FGF and 20 ng/ml EGF (herein referred to as NPC monolayer media) in the presence of 0.1, 1 or 10 ng/ml HDGF or VC (0.2 mM TRIS pH 7.5, 0.01 mM EDTA, 0.01 mM DTT, 0.1% glycerol in 1xPBS) for 1-5 DIV. 3 μg/ml BrdU (Sigma) was added to cultures for 2h before fixation.

#### D) OPC monolayer cultures:

To induce OPC formation, dissociated primary neurosphere cells were plated at 39,500 cells/cm^2^ on pre-coated coverslips as described above (section C) in SFM supplemented with 2% B27, 10 ng/ml FGF and 10 ng/ml PDGF-AA (platelet derived growth factor AA, R&D) (herein referred to as OPC growth media [GM]) and cultured for 2-3 DIV. This culture system yields ∼94% Olig2^+^ and over 60% PDGFRα^+^ OPCs with no microglia contamination (Watson *et al*., 2021). To induce OPC differentiation, media was changed on 2-3DIV to SFM devoid of growth factors and supplemented with 2% B27 and 40 ng/ml T3 (3,3',5-Triiodo-L-thyronine, Sigma) (herein referred to as OPC differentiation media [DM]). Cells were cultured for additional 3 DIV in the presence of 10 ng/ml HDGF or VC (0.2 mM TRIS pH 7.5, 0.01 mM EDTA, 0.01 mM DTT, 0.1% glycerol in 1xPBS), or in the presence of 10 ng/ml HDGF and mouse IgG (sc-3877) or anti-C23 (Nucleolin) antibody (MS-3) (sc-8031) (Chen *et al*., 2015; Lin *et al*., 2019).

### Immunocytochemistry

NPC or OPC monolayer cultures were fixed with 4% PFA for 10 min at room temperature. The cells were then permeabilized with 0.2% NP-40 in 1x PBS, blocked with 0.5% BSA, 6% normal donkey serum (Jackson ImmunoResearch) in 1x PBS, and incubated with primary antibodies (listed below) in 1⁄2 blocking buffer diluted with 1x PBS for 2h at room temperature or overnight at 4°C. After extensive washing, appropriate secondary antibodies (listed below) were added in 1x PBS for 1h at room temperature. At this point, nuclei were stained using Hoechst solution (Riodel-De Haen Ag) and coverslips were mounted in Fluoromount-G (Invitrogen), or cells were further processed for BrdU staining. Here, cultures were post-fixed with 4% PFA and then incubated with 1M HCl at 4°C for 10 min, followed by 2M HCl for 20 min at room temperature. The cells were then blocked with 1M glycine (Sigma), 1% Triton X-100 (Bio Basic), and 5% normal donkey serum (Jackson ImmunoResearch) for 30 min at room temperature. After blocking, anti-BrdU (Abcam, 1/1000 dilution) was added to the cells for 1h at room temperature or at 4°C overnight. Secondary antibodies (Donkey anti-sheep-647, Jackson ImmunoResearch, 1/500) were then added to the cells for 1h. Nuclei were stained using Hoechst and coverslips were mounted as described above.

### Immunohistochemistry (IHC)

Following perfusion as described in “Mice” section, the brains were incubated in 4% PFA at 4°C for an additional 16-24 h. The tissue was then placed into 30% sucrose (Fisher) in 1xPBS for 72h before the brains were frozen in O.C.T (optimal cutting temperature) compound (Fisher). The brains were cryosectioned coronally at 18 μm. Brain sections were re-hydrated with 1x PBS, and then blocked and permeabilized for 1 h at room temperature with 5% BSA (Jackson ImmunoResearch) and 0.3% Triton-X100 (Bio Basic) in PBS. Sections were then incubated with primary antibodies (listed below) diluted in 5% BSA in 1x PBS at 4°C overnight. Appropriate secondary antibodies (listed below) diluted in 1x PBS were applied to sections for 1h at room temperature. At this point, nuclei were stained using Hoechst solution (Riodel-De Haen Ag) and sections were mounted in Fluoromount-G (Invitrogen), or sections were further processed for BrdU staining. Here, after the incubation with the secondary antibodies, the sections were post-fixed for 10 min with 4% PFA at room temperature. The sections were then incubated at 4°C for 10 min with 1M HCl, followed by 2M HCl incubation for 10 min at room temperature and 20 min at 37°C. After extensive washing, sections were blocked with 5% normal donkey serum, 1% Triton X-100 and 1M glycine in 1X PBS and incubated with anti-BrdU (Abcam) overnight at 4°C. Donkey anti-sheep-647 antibody was added for 1 h at room temperature. Nuclei were stained using Hoechst and coverslips were mounted as described above. When antibodies raised in mouse species were used, Mouse on Mouse (M.O.M) kit (Vector Labs) was used to enhance the signal as per manufacturer’s instructions.

### Antibodies

Mouse anti-BCAS1 (Santa Cruz, 1:500, ICC and IHC, RRID: AB_10839529), mouse anti-βIII (BioLegend, 1:1000, ICC, RRID:AB_10063408), rabbit anti-βIII (BioLegend, 1:2000, RRID: AB_2564645), sheep anti-BrdU (Abcam, 1:1000, ICC, RRID:AB_302659), rabbit anti-CC3 (Millipore, 1:500, ICC, RRID: AB_91556), rabbit anti-GFAP (Dako, 1:1000, ICC, RRID: AB_10013382), rat anti-GFAP (Thermo Fisher, 1;1000, RRID:AB_2532994), chicken anti-eGFP (Abcam, 1:1000, IHC, RRID: AB_300798), mouse anti-Ki67 (BD Pharmingen, 1:300, ICC, RRID: AB_396287), rat anti-MBP (a. a. 82-87) (Millipore, 1:500, ICC, RRID: AB_94975), goat anti-PDGFRα (R&D Systems, 1:400 for IHC, 1:300 for ICC, RRID: AB_2236897), rabbit anti-PDGFRa (Santa Cruz, 1:300 for IHC, RRID: AB_631064), rabbit anti-PLP (Abcam, 1:500, ICC, RRID: AB_776593), rabbit anti-SOX2 (Cell Signalling, 1:2000, ICC, RRID: AB_2194037), goat anti-SOX2 (R&D Systems, 1:1000, IHC, RRID: AB_355110). Fluorescently labeled highly cross-absorbed secondary antibodies were purchased from Jackson ImmunoResearch and used at 1:1000 dilution. If MOM kit was used, Cy3-, DTAF-, or Cy5 conjugated streptavidin (Jackson ImmunoResearch) were used at 1:1000 dilution.

### Microscopy

Secondary neurospheres were counted using the inverted light Primovert microscope (Zeiss) and 4x objective. Only neurospheres with at least 50 cells are counted.

**Figure 2.**
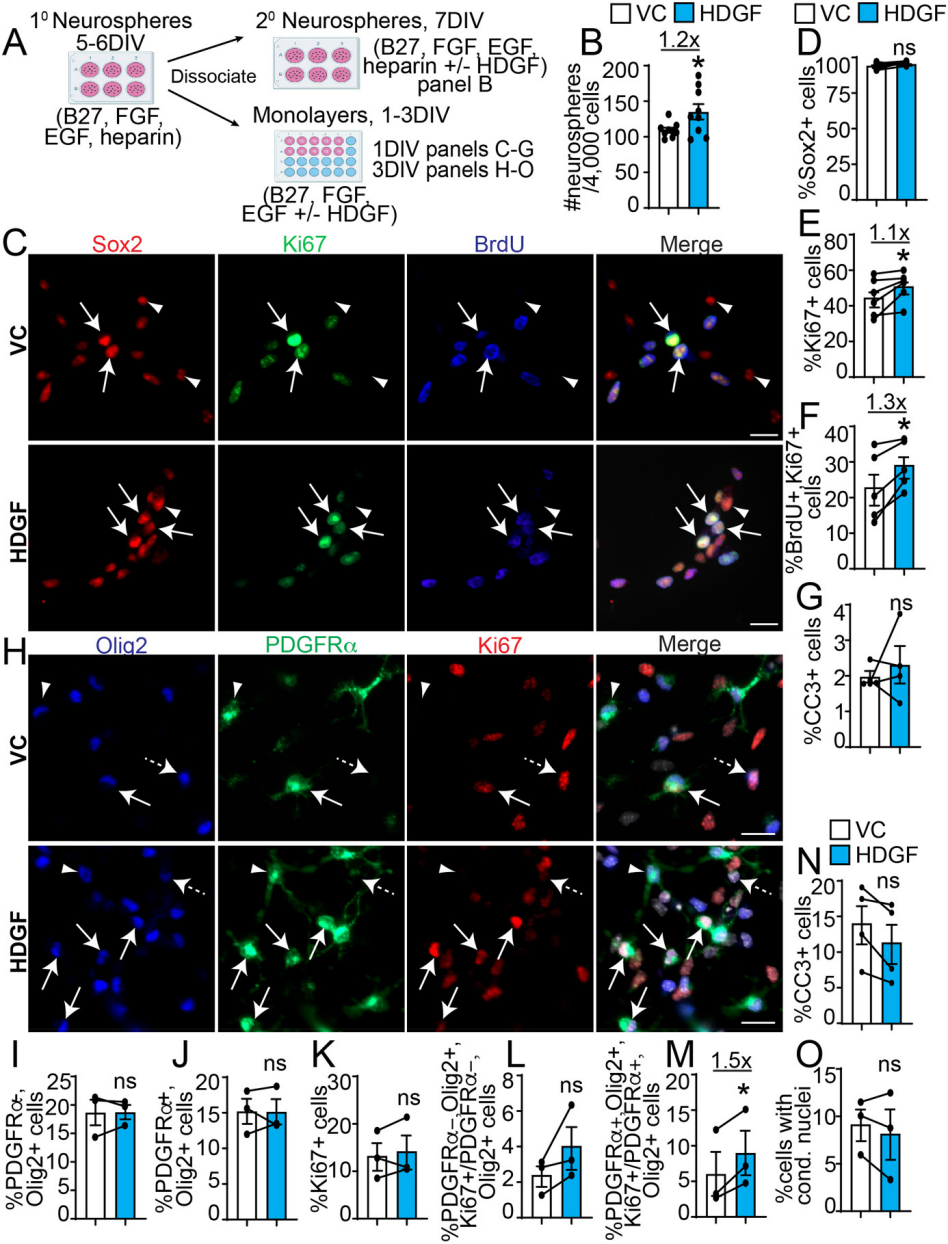
HDGF increases SVZ NPC and OPC proliferation *in vitro*. **A**. Schematic: P7 SVZ primary neurosphere cells were cultured as secondary neurospheres in media containing 2% B27, 10 ng/mL FGF, 20 ng/mL EGF and 2 μg/ml heparin sodium salt for 5DIV (**B**) or monolayers in NPC media containing 2% B27, 10 ng/mL FGF and 20 ng/mL EGF for 1 (**C-G**) or 3 (**H-O**) DIV with HDGF (10 ng/ml) or VC. **B.** Analysis of the number of secondary neurospheres seeded in VC (white bars) or 10 ng/ml HDGF (blue bars) at clonal density. Data are expressed as number of secondary neurospheres per 4,000 seeded primary neurosphere cells. *p < 0.05; n = 3 independent experiments with 3 technical replicates per experiment. **C.** Representative images of 1DIV monolayer NPCs cultured with VC (top) or 10 ng/ml HDGF (bottom) and immunostained for Ki67 (green), BrdU (blue), and Sox2 (red). Arrows indicates Ki67 + BrdU + Sox2 + cells. Arrowheads indicate Ki67-BrdU-Sox2 + cells. Cells were counterstained with Hoechst 33258 (merge panel, grey). **D-G.** Quantification of **C** for proportion of Sox2 + (**D**), Ki67 + (**E**), Ki67 + BrdU + (**F**) and CC3 + (**G**) cells in VC (white bars) or HDGF (blue bars). * p < 0.05; ns = not significant; n = 4-5 independent experiments, at least 500 cells per group per experiment. **H.** Representative images of 3DIV monolayer NPCs cultured with VC (top) or 10 ng/ml HDGF (bottom) and immunostained for Olig2 (blue), PDGFRα (green) and Ki67 (red). Please note PDGFRα images were enhanced in a non-linear way. Solid arrows indicates Olig2 + PDGFRα + Ki67 + cells, dashed arrows Olig2 + PDGFRα-Ki67 + cells, and arrowheads Olig2 + PDGFRα + Ki67- cells. Cells were counterstained with Hoechst 33258 (grey, merge panel, grey). **I-O.** Quantification of (**H**) for the proportion of PDGFRα-Olig2 + (**I**), PDGFRα + Olig2 + (**J**), Ki67 + (**K**), proliferative index of PDGFRα-Olig2 + cells (expressed as % PDGFRα-Olig2 + Ki67 + of total PDGFRα-Olig2 + cells) (**L**), proliferative index of PDGFRα + Olig2 + cells (expressed as % PDGFRα + Olig2 + Ki67 + of total PDGFRα + Olig2 + cells) (**M**), CC3 + (**N**) or cells with condensed nuclei (**O**) in VC (white bars) and 10 ng/ml HDGF (blue bars). *p < 0.05; ns = not significant. n = 3-4 independent experiments, at least 1000 cells per group per experiment. Marker + cells were expressed as % of healthy Hoechst + cells. CC3 + cells or cells with condensed nuclei were expressed as % of total Hoechst + cells. Proliferative index was expressed as % marker + Ki67 + cells over marker + cells. Scale bars are 20 µm. Error bars represent SEM. All graphs were analyzed with paired t-test, except graph in **B** was analyzed with unpaired t-test. * p < 0.05.

**Figure 1.**
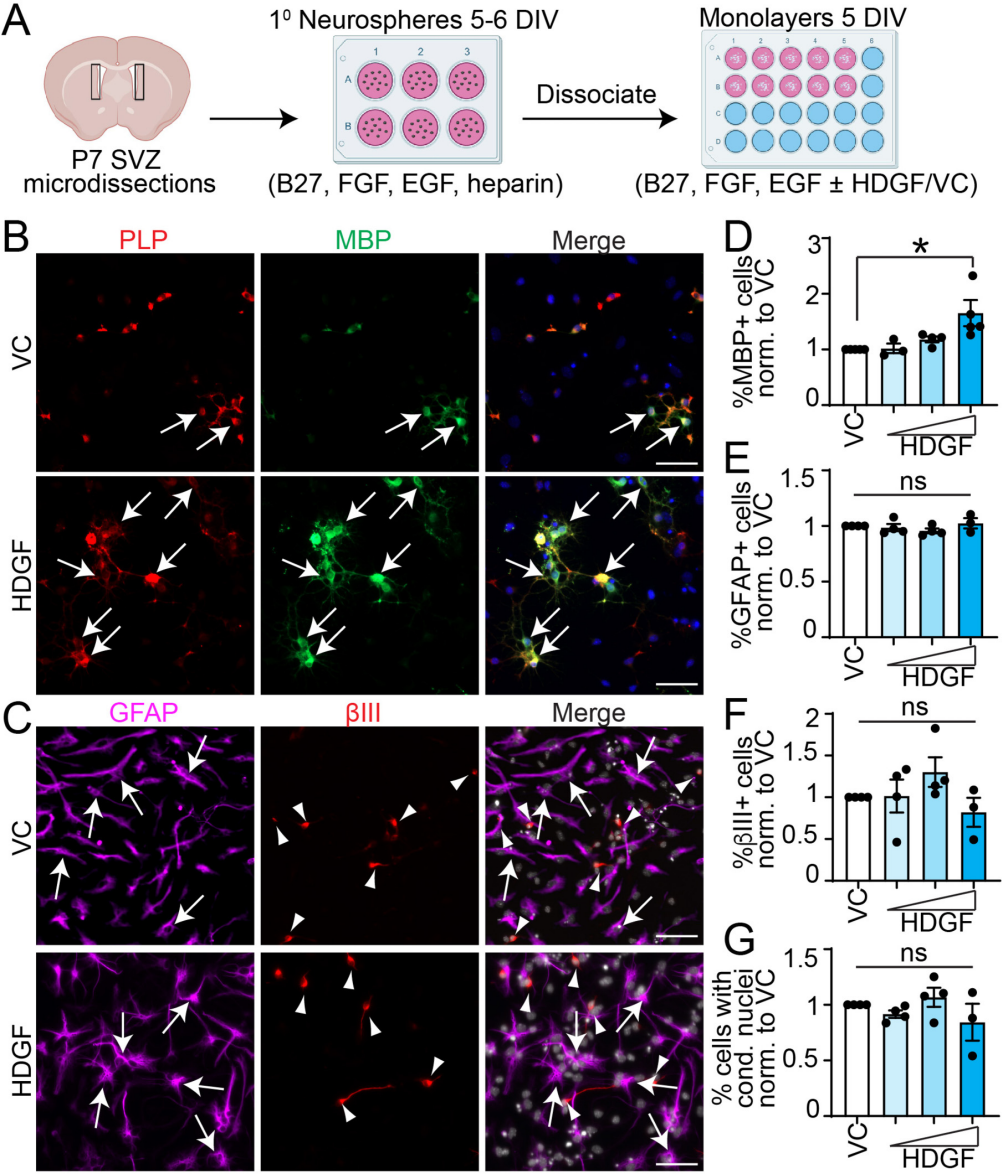
HDGF increases oligodendrocyte formation from SVZ NPCs in vitro. Please see associated Fig. S1. **A**. Schematic: primary neurospheres were generated from microdissected P7 SVZ, dissociated and cultured in media containing 2% B27, 10 ng/mL FGF, 20 ng/mL EGF and 2 μg/ml heparin sodium salt for 5-6 DIV followed by monolayer cultures in NPC media containing 2% B27, 10 ng/mL FGF and 20 ng/mL EGF for 5DIV with HDGF (0.1, 1 and 10 ng/ml) or VC (vehicle-control)**. B.** Representative images of NPCs cultured with VC (top) or HDGF (bottom) and immunostained for PLP (red) and MBP (green) (please note images were enhanced in a non-linear way). Arrows indicate marker + cells. Cells were counterstained with Hoechst 33258 (blue in merge). **C.** Representative images of NPCs cultured with VC (top) or HDGF (bottom) and immunostained for GFAP (purple), and βIII (red). Cells were counterstained with Hoechst 33258 (grey in merge). Arrows indicate GFAP + cells, and arrowheads βIII + cells. **D-G.** Quantification of **B-C** for the proportion of MBP + (**D**), GFAP + (**E**), βIII + (**F**) cells or cells with condensed nuclei (**G**) in VC (white bars) or 0.1, 1, and 10 ng/ml HDGF (blue bars). *p < 0.05, ns = not significant. n = 3-5 independent experiments, at least 1000 cells per group per replicate. Marker + cells were expressed as % of healthy Hoechst + cells. Cells with condensed nuclei were expressed as % of total Hoechst + cells. Data were normalized to VC. Scale bars are 50 µm. Error bars represent SEM. All graphs were analyzed with one-way ANOVA (p = 0.02 in D) followed by Dunnett’s multiple comparison test (* p = 0.01 in D).

**Figure 3.**
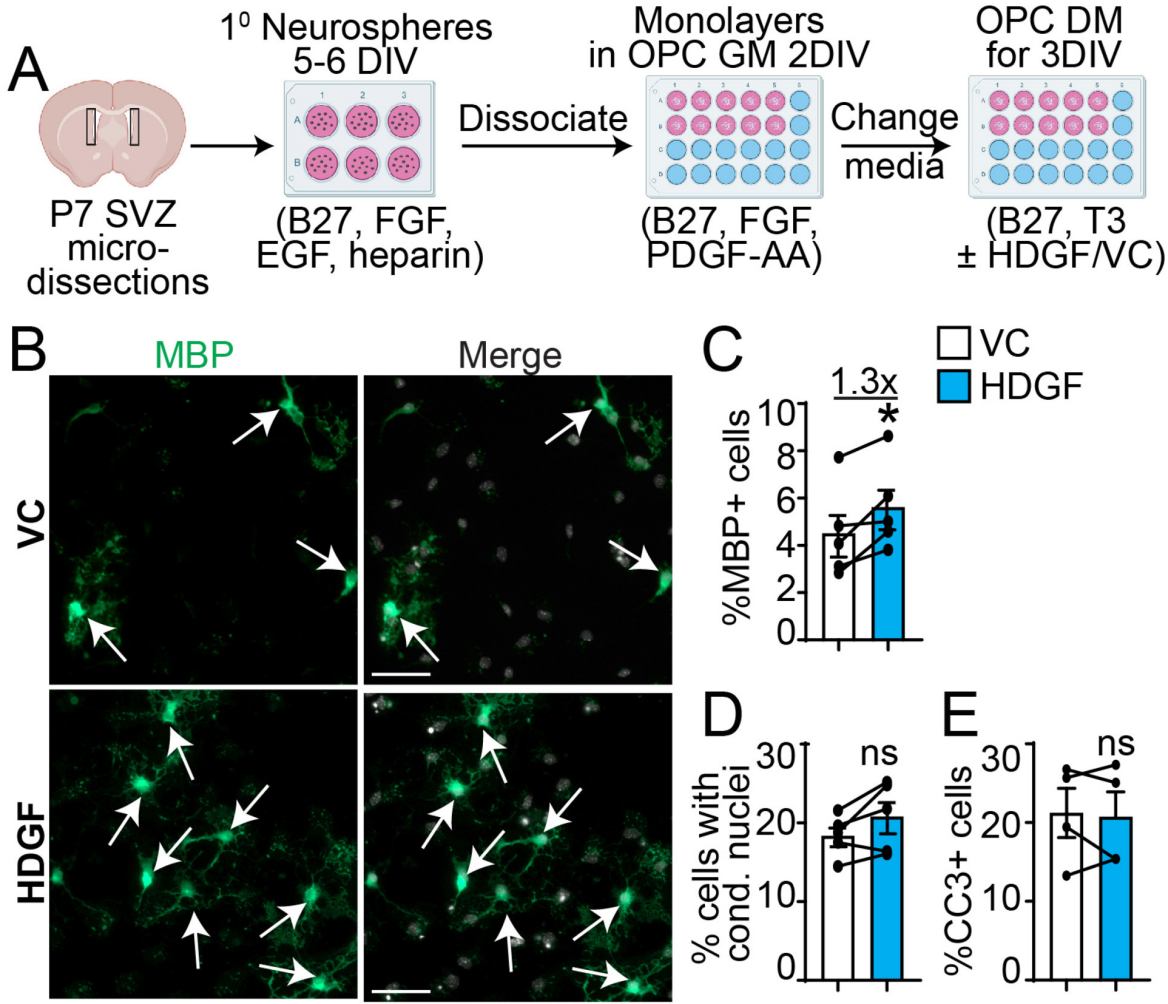
HDGF increases SVZ OPC differentiation *in vitro*. **A**. Schematic: primary neurosphere cells were generated from P7 SVZ, and cultured as monolayers in OPC growth media (GM containing 2% B27, 10 ng/mL FGF and 10 ng/mL PDGF-AA) for 2DIV, followed by treatment with OPC differentiation media (DM containing 2% B27 and 40 ng/ml T3) supplemented with VC or 10 ng/ml HDGF for 3DIV. **B.** Representative images of OPCs cultured in DM with VC (top) or HDGF (bottom) and immunostained for MBP (green). Please note MBP images were enhanced in a non-linear way. Arrows indicate marker + cells. Cells were counterstained with Hoechst 33258 (grey in merge). **C-E.** Quantification of **B** for the proportion of MBP + cells (**C**), cells with condensed nuclei (**D**) or CC3 + cells (**E**) in VC (white bars) and HDGF (blue bars). *p < 0.05; ns = not significant, n = 4-5 independent experiments, at least 500 cells per group per experiments. Marker + cells were expressed as % of healthy Hoechst + cells. CC3 + cells or cells with condensed nuclei were expressed as % of total Hoechst + cells. Scale bars are 50 µm. Error bars represent SEM. All graphs were analyzed with paired t-test.

All monolayer culture experiments as well as *in vivo* infusion experiments were captured using Zeiss Axio Imager M2 fluorescence microscope, ORCA-Flash LT sCMOS Camera, 20X objective and the Zen software (Zeiss). Cultures and *in vivo* images were imaged in a single plane for quantification. For representative *in vivo* images, single plane images or Z-stacks (taken with optical slice thickness 1 μm and stacked) are shown. Representative images showing PLP, MBP and/or PDGFRα in Figures 1B, 2H, 3B, 6D and 7D have been non-linearly enhanced. Analysis (as described below) was performed using images with linear enhancement only.

### Image Analysis and Quantification

For *in vitro* cultures, SVZ dissected and pooled from at least two pups from the same litter was considered a “biological experiment”. Secondary neurosphere experiments were analyzed from three biological experiments. Samples were plated in technical triplicates. Over 100 spheres were analyzed per well, per condition and per experiment. In monolayer culture experiments, 5 random fields of view from technical duplicates per biological experiment were captured with a 20X objective. At least 500-1,000 cells from each treatment and 3-5 biological experiments were counted. Cells with condensed nuclei or CC3^+^ cells are presented as relative to total (sum of cells with condensed and healthy) nuclei. For all remaining monolayer culture experiments, results are presented as marker^+^ cells relative to total healthy nuclei. Proliferation index is presented as %Ki67^+^marker^+^ cells over total marker^+^ cells.

For *in vivo* infusion experiments, lateral or dorsal SVZ and/or neighbouring corpus callosum (CC) surrounding the infused ventricle was tile-imaged using 20X objective as indicated in figure legends. Areas of interest were identified with Hoechst staining. The results are presented as number of marker^+^ cells per section, percent marker^+^YFP^+^ cells relative to total YFP^+^ cells, or as proliferation index (percent marker^+^Ki67^+^ or marker^+^BrdU^+^ cells relative to total marker^+^ cells). 5-10 anatomically matched sections per brain were analyzed from at least 3 mice per treatment across two independent litters. At least 250 cells per dorsal SVZ and CC or at least 1,000 cells per lateral SVZ were counted.

Cells were counted from digital images using Zen or Fiji (Schindelin *et al*., 2012). Representative images were processed in Photoshop CC 2018 and figures were prepared in Adobe Illustrator CC 2018. Biorender was used to generate schematics in all figures.

Sample sizes (n) are indicated in figure legends and correspond to the number of biological replicates analyzed (Table 1). All primary culture experiments were performed in technical duplicates or triplicates from at least 3 different litters. Each *in vitro* data point from monolayer cultures in figures corresponds to an average value from 2-3 technical replicates. Each *in vitro* data point from secondary neurosphere cultures corresponds to a technical replicate from three biological experiments. All *in vivo* data are from at least 6 mice (at least 3 mice per treatment) from at least two independent litters. Each *in vivo* datapoint in figures corresponds to each individual mouse. All data are presented as mean ± SEM.

### Statistical Analysis

For two group comparisons, two-tailed paired (*in vitro* monolayer culture datasets) or unpaired (secondary neurosphere and *in vivo* datasets) student’s t-tests or multiple t-test (*in vivo* datasets) were used to assess statistical significance between means, where a p-value < 0.05 was considered significant. For three or more group comparisons one-way ANOVA was followed by Dunnett’s or Tukey multiple comparisons test. In all cases, Prism (version 8.0.2) was used. Number of experiments and statistical information are stated in the corresponding figure legends. In figures, asterisks denote statistical significance marked by *, p < 0.05; **, p < 0.01.

## Results

### HDGF Increases Oligodendrocyte Genesis from SVZ NPCs *in Vitro*

To determine whether HDGF can modulate SVZ NPC fate, we cultured cells microdissected from postnatal day (P) 7 SVZ tissue as primary neurospheres, which were then incubated as adherent cultures for 5 days *in vitro* (DIV) in the presence of B27, EGF and FGF as well as VC or HDGF (0.1-10 ng/ml) (Figure 1A). This culture system yields highly enriched Nestin^+^,Sox2^+^ NPCs (∼98% enrichment) and allows studying the effect of external ligands on SVZ NPC proliferation and differentiation (Watson *et al*., 2021). On day 5 of SVZ monolayer culture, when neurons and glial cells arise (Watson *et al*., 2021), we observed a statistically significant (p = 0.024) ∼1.65 fold increase in the formation of MBP^+^ oligodendrocytes in the presence of HDGF in a concentration-dependent manner when compared to VC (Figure 1B, D). As NPCs also differentiate into neurons and astrocytes (Obernier and Alvarez-Buylla, 2019), it is possible HDGF enhances SVZ NPC *in vitro* oligodendrocyte genesis at the expense of neurons or astrocytes. Analysis of GFAP^+^ (Glial Fibrillary Acidic Protein) astrocytes and βIII^+^ neurons revealed no changes between VC and HDGF groups (Figure 1C, E-F). Finally, exogenous HDGF has been shown to increase neuron survival (Zhou *et al*., 2004). We thus considered that HDGF may have an effect on cell death. Quantification of condensed nuclei, which is a well-established method of measuring apoptosis (Mandelkow *et al*., 2017), or cells positive for cleaved caspase 3 (CC3) staining revealed that HDGF did not affect apoptosis or the number of cells with condensed nuclei (Figs. 1G, S1).

Therefore, exogenous HDGF specifically increases oligodendrocyte genesis from SVZ NPCs *in vitro*.

### HDGF Increases SVZ NPC and OPC Proliferation *in Vitro*

We next asked whether HDGF exerts a pro-oligodendrogenic effect by stimulating NPC and/or OPC proliferation. First, we cultured NPCs as secondary neurospheres, which allows NPC proliferation, at clonal density (Coles-Takabe *et al*., 2008) (Figure 2A). We observed a statistically significant (p = 0.038) ∼1.2-fold increase in the number of secondary neurospheres cultured in the presence of HDGF when compared to VC (Figure 2B). We then sought to corroborate these results in NPC monolayer cultures, which allow simultaneous proliferation and differentiation (Watson *et al*., 2021). Primary neurosphere SVZ cells were incubated as adherent cells for 20-24h (1DIV) in VC or HDGF. 2h before harvest, cells were pulsed with BrdU. Resulting cultures were fixed and stained with antibodies specific for proliferating cells (Ki67 and BrdU) and NPCs (Sox2 [Bylund *et al*., 2003; Graham *et al*., 2003; Ahmed, 2009]) (Figure 2C). Our results demonstrate 1DIV NPC cultures were mostly comprised of Sox2^+^ NPCs (∼94% in VC and ∼95% in HDGF) (Figure 2D). In these cultures, exogenous HDGF exhibited a statistically significant (p < 0.05) ∼1.1-fold increase in Ki67^+^ cells and ∼1.3 fold increase in Ki67^+^BrdU^+^ cells without any changes in the proportion of CC3^+^ cells (Figure 2E-G).

To test the effect of HDGF on OPC proliferation, we analyzed NPC monolayer cultures on 3DIV, when we observed ∼15% PDGFRα^+^ (Platelet Derived Growth Factor Receptor α) OPCs (Figure 2H, J), in agreement with (Watson *et al*., 2021). While we did not observe any changes in the proportion of total Ki67^+^, PDGFRα^−^Olig2^+^ or PDGFRα^+^Olig2^+^ cells (Figure 2I-K), the proliferative index of PDGFRα^+^Olig2^+^ cells (%PDGFRα^+^Olig2^+^Ki67^+^ / PDGFRα^+^Olig2^+^ cells) was significantly (p = 0.03) increased by ∼1.5 fold in the presence of HDGF (Figure 2M). Notably, there was an increasing trend in the proliferative index of PDGFRα^−^Olig2^+^ cells (% PDGFRα^−^Olig2^+^Ki67^+^/PDGFRα^−^Olig2^+^ cells), although it did not reach statistical significance (p = 0.2) (Figure 2L). Similar to 1DIV and 5DIV data (Figs. 1G, S1 and 2G), HDGF did not affect the proportion of cells containing condensed nuclei or proportion of CC3^+^ cells on 3DIV (Figure 2N-O).

Together, these data show that HDGF increases SVZ NPC and PDGFRα + OPC proliferation *in vitro*. However, despite the HDGF-induced increase in OPC proliferation (Figure 2M), there was no increase in the total proportion of OPCs (Figure 2J). Thus, we predicted HDGF may also increase OPC differentiation into oligodendrocytes.

### HDGF Increases SVZ OPC Differentiation *in Vitro*

To test the effect of HDGF on OPC differentiation, we cultured primary neurosphere-derived cells in OPC growth media (GM) containing FGF and PDGF-AA. This culture protocol results in robust (∼94%) enrichment of SVZ OPCs (Watson *et al*., 2021). At this point, the media was switched to differentiation media (DM) containing thyroid hormone T3, a potent inducer of oligodendrocyte differentiation (Bhat *et al*., 1979), and VC or HDGF (Figure 3A). Figure 3B-E shows that addition of exogenous HDGF led to a statistically significant (p = 0.03) ∼1.3 fold increase in MBP^+^ oligodendrocytes without any changes in the proportion of CC3 + cells or cells containing condensed nuclei. Therefore, in addition to enhancing SVZ precursor proliferation, HDGF increases OPC differentiation into oligodendrocytes.

### HDGF Does not Affect NPC Proliferation *in Vivo*

**Figure 4.**
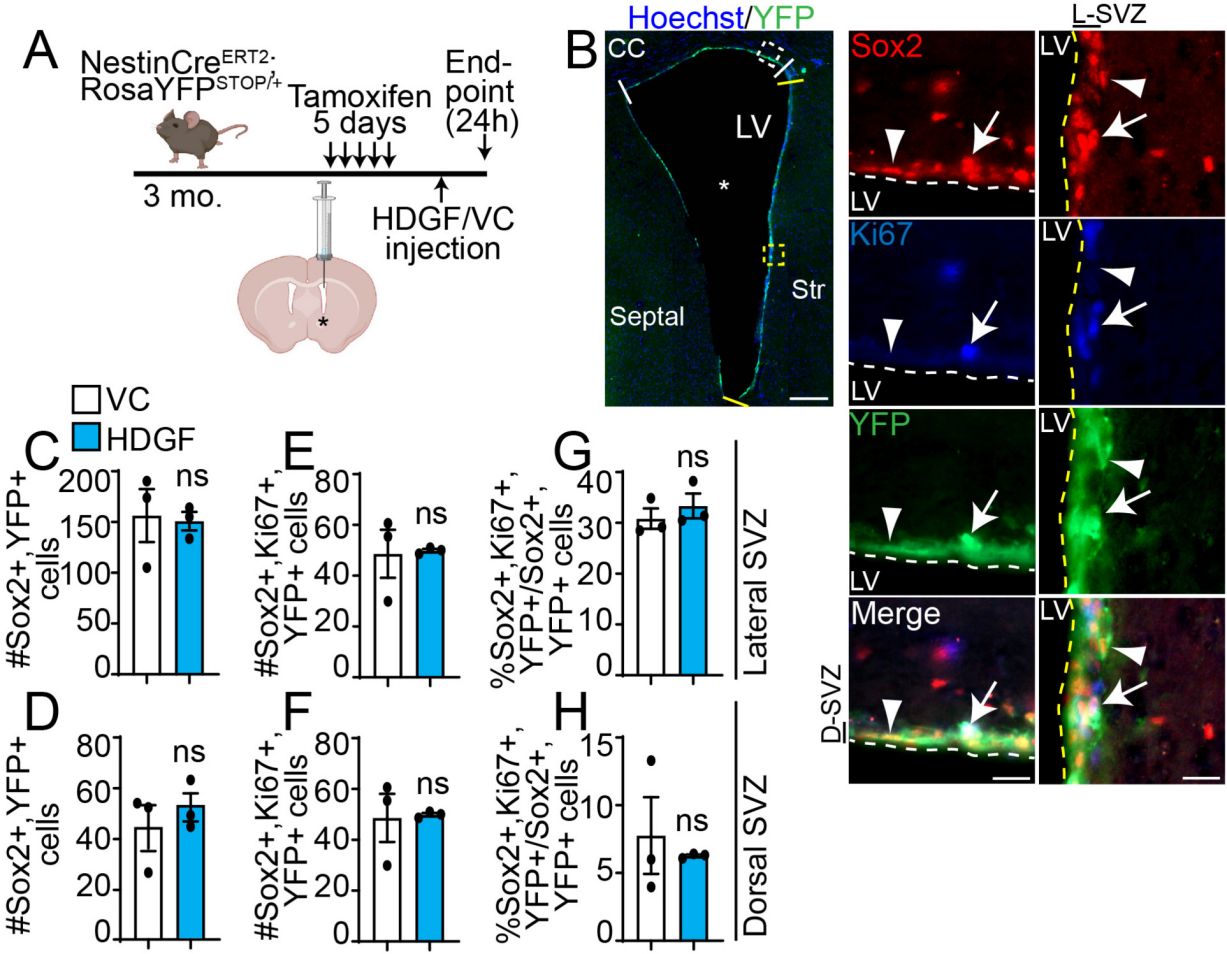
HDGF does not affect SVZ NPC proliferation *in vivo*. Please see associated Fig. S2. **A**. Schematic: 3 month old NestinCre^ERT2^;RosaYFP^STOP/+^ NPC lineage tracing mice were injected with tamoxifen for 5 days. 72h later, HDGF or VC was injected once into the lateral ventricle (LV, indicated by asterisk) through an ICV surgery. Mice were euthanized 24h after ICV injection. Dorsal and lateral SVZ (demarcated by white and yellow lines, respectively, in **B**) was analyzed in **C-H**. **B**. Representative tiled image (left) of lateral ventricle (asterisk) analyzed 24h after infusion with VC and stained for YFP (green) and counter-stained with Hoechst 33258 (blue). White and yellow hatched insets shown in the tiled image illustrate dorsal SVZ (D-SVZ, left column) and lateral SVZ (L-SVZ, right column), respectively. Sox2 is in red, Ki67 in blue and YFP in green. Arrows designate Sox2 + Ki67 + GFP + cells, and arrowhead designates a Sox2 + Ki67-YFP + cell. White and yellow hatched lines in insets demarcate SVZ boundary. **C-H**. Quantification of (**B**) for number of Sox2 + YFP + cells (**C,D**), and Sox2 + YFP + Ki67 + cells (**E,F**) expressed as an average number of marker + cells in dorsal SVZ and lateral SVZ per section. Proliferative index of Sox2 + YFP + NPCs in (**G,H**) is expressed as percent Sox2 + YFP + Ki67 + cells relative to total Sox2 + YFP + cells. Data from lateral SVZ are in **C,E,G** and from dorsal SVZ in **D,F,H**. ns = not significant; n = 3 mice from 2 independent litters. Scale bars are 200 µm in B, tiled image (left column), and 20 µm in B, insets (right columns). Error bars represent SEM. All graphs were analyzed with unpaired t-test. CC = corpus callosum; D-SVZ = dorsal SVZ; L-SVZ = lateral SVZ; LV = lateral ventricle; Str = striatum.

To test whether the pro-proliferative effect of exogenous HDGF is preserved *in vivo*, we infused HDGF or VC into the brain lateral ventricle of adult NPC lineage tracing mice (NestinCre^ERT2^;RosaYFP^STOP/+^) (Figure 4A). Here, recombination and YFP (yellow fluorescent protein) expression was induced in Nestin^+^ NPCs and their progeny with tamoxifen injections. 72h after the last tamoxifen injection, over 99% of YFP + cells in the dorsal and lateral SVZ are also Sox2 + (marker of NPCs in the SVZ [Wang *et al*., 2010; Remaud *et al*., 2013]), and over 99% of Sox2 + cells are YFP + (Figs. 4B, S2 & data not shown), in agreement with (Storer *et al*., 2018). At this time point, intracerebroventricular (ICV) infusion of HDGF or VC was performed. First, we analyzed lateral and dorsal SVZ NPC proliferation 24h after a single ICV injection. In contrast to our *in vitro* results (Figure 2E-F), exogenous HDGF did not affect the number of Sox2^+^Ki67^+^YFP^+^ cells (Figure 4E, F), or the proliferative index of Sox2^+^YFP^+^ NPCs (% Sox2^+^Ki67^+^YFP^+^/Sox2^+^YFP^+^ cells) (Figure 4G, H) in lateral or dorsal SVZ. HDGF also did not alter the total number of Sox2^+^YFP^+^ cells (Figure 4C, D).

**Figure 5.**
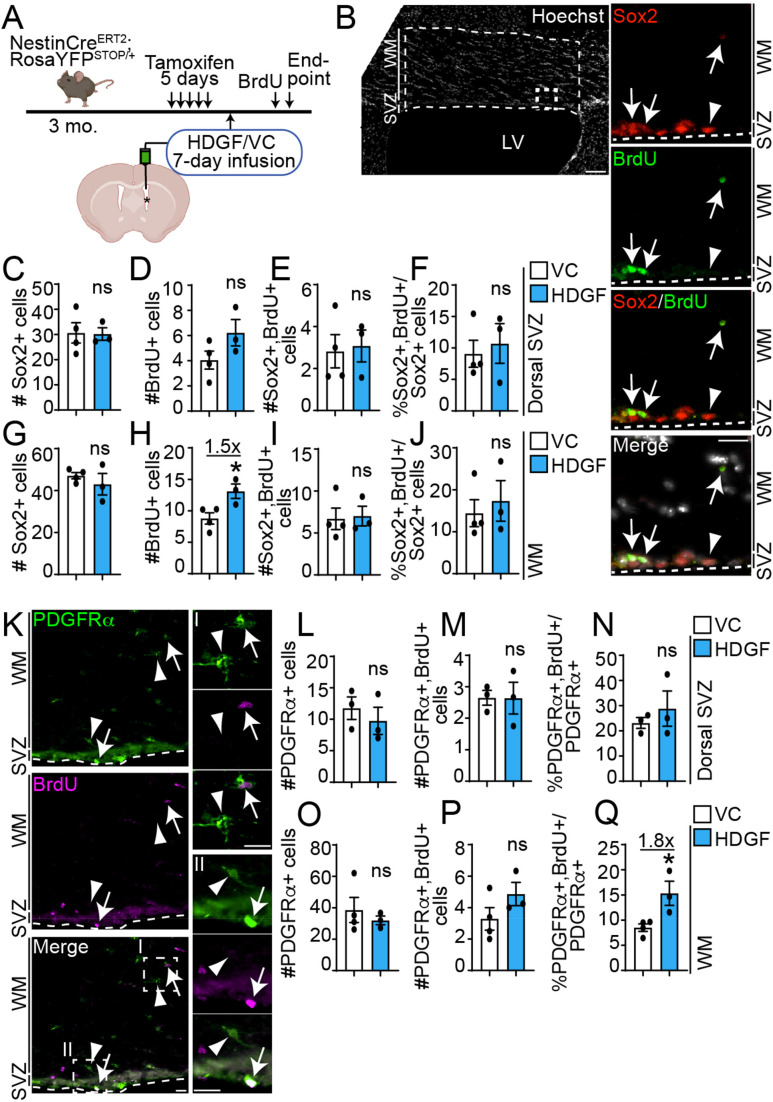
HDGF increases OPC proliferation *in vivo*. **A**. Schematic: 3 month old NestinCre^ERT2^;RosaYFP^STOP/+^ NPC lineage tracing mice were injected with tamoxifen for 5 days. 72h later, ICV surgery was performed to infuse HDGF or VC into the lateral ventricle (indicated by asterisk) for 7 days via osmotic mini-pumps. BrdU was injected 24h before euthanasia. **B**. Representative image of 7-day VC-infused ventricle stained with Hoechst 33258 (grey) (left column). Hatched inset is shown at a higher magnification in the right column. Areas (SVZ and WM [white matter]) between hatched lines were analyzed in **C-J**. Sox2 is in red, and BrdU in green. Arrows indicate Sox2 + BrdU + cells, and arrowhead indicates a Sox2 + BrdU- cell. Hatched lines in insets demarcate SVZ boundary. **C-J**. Quantification of (**B**) for number of Sox2 + cells (**C,G**), BrdU + cells (**D,H**), Sox2 + BrdU + cells (**E,I**) expressed as an average number of marker + cells per dorsal SVZ per section. Proliferative index of Sox2 + NPCs is in (**F,J**) and is expressed as percent Sox2 + BrdU + cells relative to total Sox2 + cells. Data from dorsal SVZ are in **C-F** and from white matter in **G-J**. **K**. Representative image of a 7-day HDGF-infused ventricle stained with PDGFRα (green) and BrdU (magenta). Hatched insets are shown at a higher magnification in the right column (inset I is from WM and inset II is from SVZ). Arrows and arrowheads indicate PDGFRα + BrdU + and PDGFRα + BrdU- OPCs. Hatched lines demarcate SVZ boundary. **L-Q.** Quantification of (**K**) for number of PDGFRα + cells (**L,O**), PDGFRα + BrdU + cells (**M,P**) expressed as an average number of marker + cells per dorsal SVZ or WM per section. Proliferative index of PDGFRα + OPCs is in (**N,Q**) and is expressed as percent PDGFRα + BrdU + cells relative to total PDGFRα + cells. Data from dorsal SVZ are in **L-N** and from white matter in **O-Q**. *p < 0.05. ns = not significant; n = 3-4 mice from 2 independent litters. Scale bars are 100 µm in B, left column (tiled image) and 20 µm in B, right columns (insets) and K. Error bars represent SEM. All graphs were analyzed with unpaired t-test. LV = lateral ventricle.

We then hypothesized that HDGF may modulate SVZ NPC proliferation with a longer infusion duration. To test this, we infused HDGF or VC into the lateral ventricle of NPC lineage tracing mice for 7-days via osmotic mini-pumps (Figure 5A). To label proliferating cells, BrdU was intraperitoneally injected 24h before euthanasia. As dorsal SVZ NPCs are known to migrate into the adjacent corpus callosum (white matter [WM] tracts) as undifferentiated NPCs and/or committed oligodendroglial cells (Brousse *et al*., 2021; Cebrian Silla *et al*., 2021), we focused on these regions (dorsal SVZ and WM, Figure 5B) to characterize the proliferation of Sox2^+^ cells. Since not all cells in the adjacent corpus callosum are migrated SVZ YFP^+^ cells, and a very small amount of cells proliferate in this area (Figure 5D-E, H-I), we extended our analysis to total Sox2 + cells for a more robust quantification. Similar to the 24h analysis (Figure 4), exogenous HDGF did not affect the number of Sox2^+^ cells in the dorsal SVZ (Figure 5C) or WM after a 7-day infusion (Figure 5G). However, there was a trending increase in the number of BrdU^+^ cells in the dorsal SVZ, and a statistically significant (p = 0.03) ∼1.5-fold increase in WM BrdU^+^ cells in the presence of HDGF when compared to VC (Figure 5D, H). The increase in BrdU^+^ cells was not due to an increase in Sox2^+^ cell proliferation since the number of Sox2^+^BrdU^+^ or the proliferative index of Sox2^+^ cells (% Sox2^+^BrdU^+^/Sox2^+^ cells) was not different between HDGF and VC (Figure 5E-J).

Therefore, exogenous HDGF does not affect Sox2^+^ precursor cell proliferation *in vivo*.

### HDGF Increases OPC Proliferation *in Vivo*

The ability of HDGF to increase the total number of BrdU^+^ cells in the white matter (WM) lining dorsal to the infused ventricle with a 7-day infusion protocol (Figure 5H) led us to hypothesize that it may affect OPC proliferation, as indicated by our *in vitro* analysis (Figure 2M). Only a very small number of adult OPCs proliferate (Figure 5M, P). Thus, to make our analysis more robust, we analyzed the proportion of total OPCs, irrespective of overlap with the YFP signal. Sections from brains infused for 7 days with HDGF or VC were immunostained for PDGFRα and BrdU (Figure 5K). The HDGF infusion did not modulate the number of PDGFRα^+^ or PDGFRα^+^BrdU^+^ OPCs in the dorsal SVZ or WM. However, the proliferative index of OPCs (%PDGFRα^+^BrdU^+^/PDGFRα^+^ cells) was statistically significantly (p = 0.026) increased by ∼1.8-fold in WM, but not in the dorsal SVZ in the presence of HDGF (Figure 5L-Q). These data support our *in vitro* observations (Figure 2M) and show HDGF increases OPC proliferation *in vivo*.

### HDGF Increases Oligodendrocyte Genesis from SVZ NPCs *in Vivo*

**Figure 6.**
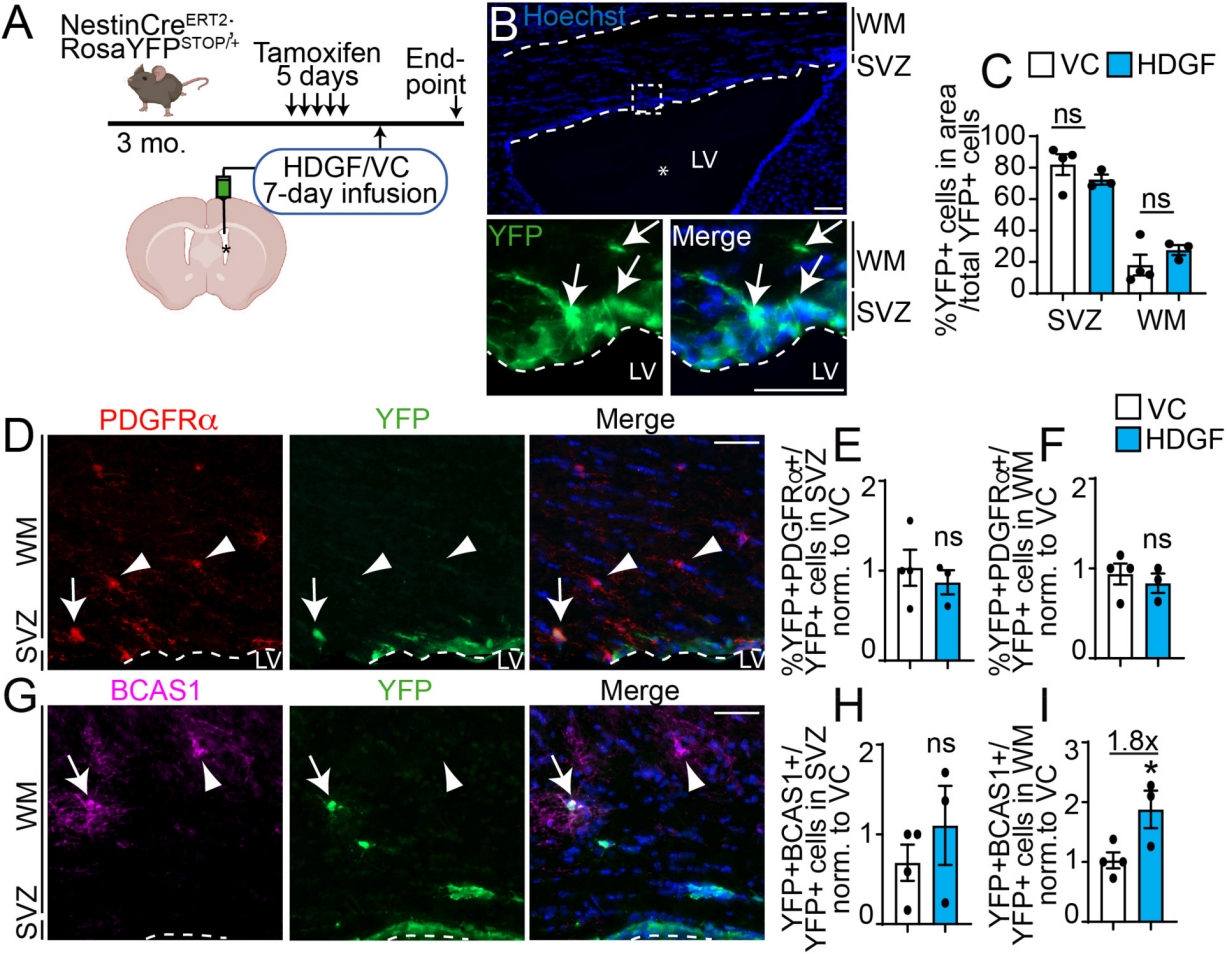
HDGF enhances oligodendrocyte genesis from SVZ NPCs *in vivo*. **A**. Schematic: 3 month old NestinCre^ERT2^;RosaYFP^STOP/+^ NPC lineage tracing mice were injected with tamoxifen for 5 days. 72h later, ICV surgery was performed to infuse HDGF or VC into the lateral ventricle (LV, indicated by asterisk) for 7 days via osmotic mini-pumps. Corpus callosum lining dorsally to the infused ventricles was captured and analyzed in **B-I**. **B.** Representative image of dorsal SVZ and white matter (WM) lining HDGF-infused ventricle (top). Hatched box from top shown at higher magnification in the bottom panel. YFP signal was amplified with antibody specific to YFP, counterstained with Hoechst 33258 to visualize nuclei (blue). Area analyzed in **C-I** is indicated by the dashed borders. LV indicates lateral ventricle, asterisk indicates infused ventricle. **C.** Proportion of YFP + cells in SVZ or WM from **(B)** is expressed as percent of total YFP + cells in both areas. **D.** Representative image of corpus callosum lining the ventricle infused with HDGF and immunostained for PDGFRα (red) and YFP (green). Please note PDGFRα images were enhanced in a non-linear way. Sections were counterstained with Hoechst 33258 (blue in merge panel). Arrow indicates PDGFRα + YFP + cells, and arrowheads indicate PDGFRα + YFP- cells. Dashed line indicates SVZ boundary and LV indicates lateral ventricle. **E-F.** Quantification of (**D**) for PDGFRα + YFP + cells in the SVZ (**E**) or WM (**F**) expressed as percent of total YFP + cells in each region of interest and normalized to VC. **G.** Representative image of corpus callosum lining the ventricle infused with HDGF and immunostained for BCAS1 (magenta) and YFP (green). Arrow indicates BCAS1 + YFP + cell, and arrowhead indicates BCAS1 + YFP- cell. Sections were counterstained with Hoechst 33258 (blue in merge panel). Dashed line indicates SVZ boundary and LV indicates lateral ventricle. **H-I.** Quantification of (**G**) for BCAS1 + YFP + cells in the SVZ (**H**) and WM (**I**) expressed as percent of total YFP + cells in the region of interest and normalized to VC. *p < 0.05. ns = not significant; n = 3-4 mice from 2 independent litters. Scale bars are 100 µm in B (top panel) and 50 µm in all other panels. Error bars represent SEM. All graphs were analyzed with unpaired t-test except graph in (**C**) was analyzed with multiple t-test.

To test whether HDGF increases OPC commitment and/or oligodendrocyte differentiation from SVZ NPCs *in vivo*, we performed lineage tracing analysis of YFP^+^ cells in the NestinCre^ERT2^;RosaYFP^STOP^ animals infused with HDGF or VC for 7 days (Figure 6A). This duration is sufficient for SVZ NPCs to form oligodendrocytes *in vivo* (Watson *et al*., 2021). Since dorsal SVZ NPCs give rise to oligodendroglial cells (Ortega *et al*., 2013; Cebrian Silla *et al*., 2021), we focused our analysis on the dorsal SVZ and white matter (WM) tracts adjacent to the infused ventricle (Figure 6B).

First, we determined whether HDGF infusion led to a different YFP^+^ cell distribution between the SVZ and white matter. Figure 6C showed that while there was a trending decrease and increase in the proportion of YFP^+^ cells in the dorsal SVZ and white matter tracts, respectively, these data did not reach statistical significance. Therefore, exogenous HDGF most likely does not regulate YFP^+^ cell migration. We then asked about the identity of YFP^+^ cells in these regions. First, we counter-stained sections with YFP- and PDGFRα-specific antibodies to detect newly born OPCs (Figure 6D). The analysis showed HDGF did not affect the formation of YFP^+^PDGFRα^+^ OPCs in the dorsal SVZ or WM (Figure 6E-F), in agreement with our *in vitro* results (Figure 2J). Next, we counter-stained sections with antibodies raised against YFP and BCAS1 (breast carcinoma amplified sequence 1 [Fard *et al*., 2017; Ishimoto *et al*., 2017]) to identify *de novo* oligodendrocytes (Figure 6G). While HDGF did not affect the formation of YFP^+^BCAS1^+^ oligodendrocytes in the dorsal SVZ (Figure 6H), it increased the proportion of newborn YFP^+^BCAS1^+^ oligodendrocytes in the white matter by ∼1.8 fold compared to VC (Figure 6I).

Therefore, exogenous HDGF increases oligodendrocyte formation from SVZ NPCs *in vivo*.

### HDGF Functions Through an Unidentified Receptor

**Figure 7.**
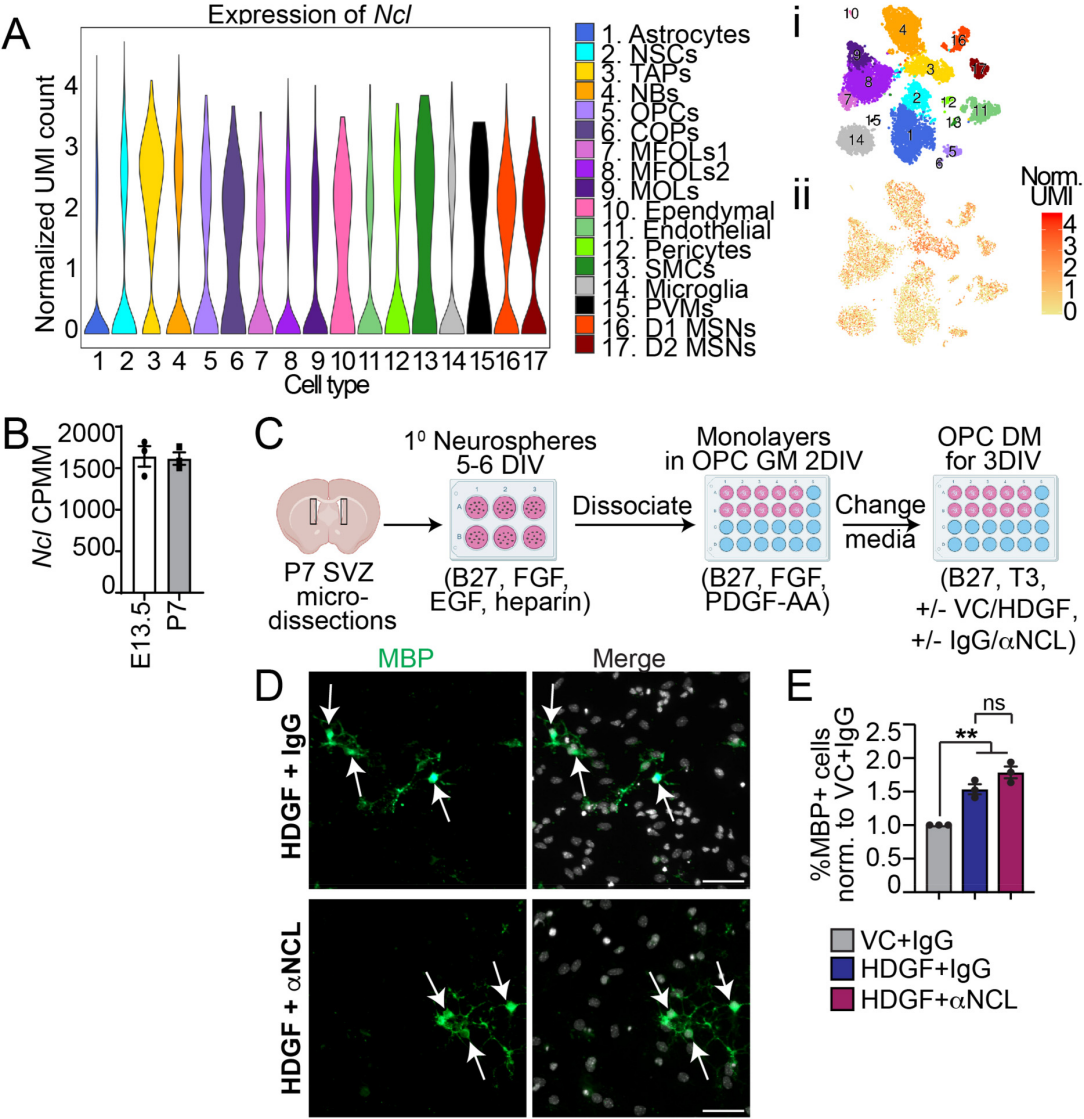
HDGF does not regulate oligodendrocyte genesis via NCL. **A**. A violin plot of *Ncl* mRNA expression extracted from a single cell adult SVZ RNA-sequencing dataset (Zywitza *et al*., 2018) (https://shiny.mdc-Berlin.de/SVZapp/). i and ii demonstrate tSNE map of SVZ cell clusters (i) and *Ncl* expression in these clusters (ii). UMI = unique molecular identifier. NSCs = neural stem cells; TAPs = transient amplifying progenitors; NBs = neuroblasts; OPCs = oligodendrocyte progenitor cells; COPs = differentiation-committed oligodendrocyte precursors; MFOLs = myelin forming oligodendrocytes; MOLs = mature oligodendrocytes; SMCs = smooth muscle cells; PVMs = perivascular macrophages; MSNs = medium spiny neurons. **B.** *Ncl* mRNA expression (CPMM = counts per million) extracted from bulk RNA sequencing of purified E13.5 and P7 brain PDGFRα^+^ OPCs (Marques *et al*., 2018) (https://castelobranco.shinyapps.io/OPCsinglecell2017/). **C.** Schematic: primary neurosphere cells were generated from P7 SVZ, dissociated and cultured as monolayers in OPC growth media (GM containing 2% B27, 10 ng/mL FGF and 10 ng/mL PDGF-AA) for 2DIV, followed by treatment with OPC differentiation media (DM containing 2% B27 and 40 ng/ml T3) supplemented with VC and 5 μg/ml IgG or 10 ng/ml HDGF and 5 μg/ml IgG or anti-NCL for 3DIV. **D.** Representative images of OPCs cultured in DM with HDGF and IgG (top) or HDGF and anti-NCL (bottom) and immunostained for MBP (green). Please note MBP images were enhanced in a non-linear way. Arrows indicate marker + cells. Cells were counterstained with Hoechst 33258 (grey in merge). **E.** Quantification of **D** for the proportion of MBP + cells in HDGF and anti-NCL (red bar) and HDGF and IgG (dark blue bar) normalized to VC and IgG (grey bar). ns = not significant, n = 3 independent experiments, at least 500 cells per group per experiments. Marker + cells were expressed as % of healthy Hoechst + cells. Scale bars are 50 µm. Error bars represent SEM. All graphs were analyzed with paired t-test, except graph in E was analyzed with one-way ANOVA (p = 0.0015) followed by Tukey multiple comparison test (p < 0.01). ns = not significant.

Thus far, only one receptor, nucleolin (NCL), has been identified to interact with and mediate the uptake of HDGF in hepatoma cells (Chen *et al*., 2015; Lin *et al*., 2019). An adult SVZ cell single cell RNA-sequencing dataset (Zywitza *et al*., 2018) (https://shiny.mdc-berlin.de/SVZapp/) shows that NCL is expressed in a variety of SVZ niche cells, including transit-amplifying cells (TAPs) and OPCs (Figure 7A). Bulk RNA-sequencing confirms NCL is expressed in PDGFRα^+^ OPCs isolated from the E13.5 and P7 brain (Figure 7B) (Marques *et al*., 2018) (https://castelobranco.shinyapps.io/OPCsinglecell2017/). To test whether HDGF could increase oligodendrocyte genesis via NCL, SVZ OPCs were differentiated in the presence of VC with non-specific isotype-matched IgG, or HDGF with non-specific isotype-matched IgG or NCL-specific function blocking antibody, which was previously used at 5-10 μg/ml to block HDGF uptake in cancer cells (Chen *et al*., 2015; Lin *et al*., 2019) (Figure 7C). Since 10 μg/ml concentration was toxic to the SVZ NPCs and OPCs (data not shown), we co-treated cells with 5 μg/ml of IgG or anti-NCL. At 3DIV, the proportion of MBP^+^ cells in IgG and HDGF-exposed SVZ OPC was increased when compared to IgG and VC, as expected (Figure 7D-E). However, the proportion of MBP + cells in HDGF-exposed cultures did not differ between IgG and anti-NCL groups (Figure 7E). Treatment of SVZ NPCs with HDGF and anti-NCL also did not show a decrease in the proportion of MBP^+^ or BCAS1^+^ oligodendrocytes when compared to IgG (data not shown). Therefore, HDGF may function through a different, yet unidentified receptor on SVZ NPCs and/or OPCs.

## Discussion

Our data demonstrate a novel role for exogenous HDGF in postnatal and adult SVZ oligodendrocyte genesis. We have shown that exogenous HDGF increases SVZ NPC and OPC proliferation, as well as OPC differentiation *in vitro*. *In vivo* infusion of exogenous HDGF increases oligodendrocyte genesis from adult SVZ NPCs and OPC proliferation. Our results suggest HDGF is a candidate molecule for modulation of SVZ precursor cell fates during oligodendrocyte genesis.

SVZ NPC fates are in large part regulated by the surrounding environment and neighbouring cells, such as the choroid plexus and cerebral spinal fluid (Silva-Vargas *et al*., 2016), endothelial cells (Paredes *et al*., 2021), microglia (Matarredona *et al*., 2018) and axonal innervation (Paul *et al*., 2017). Not surprisingly, intracerebral or intraventricular administration of various ligands, such as EGF, pigment epithelium-derived factor (PEDF), FGF-2, Sonic Hedgehog (Shh) and fractalkine (CX3CL1) increases oligodendroglial cell genesis from SVZ NPCs (Loulier *et al*., 2006; Aguirre *et al*., 2007; Sohn *et al*., 2012; Watson *et al*., 2021 and reviewed in Gonzalez-Perez and Alvarez-Buylla, 2011; Xapelli *et al*., 2014). Here, we extend the repertoire of soluble molecules capable of enhancing SVZ oligodendroglial cell genesis, and show HDGF instructs SVZ NPCs to form oligodendrocytes both in culture and *in vivo*.

Our results show exogenous HDGF does not enhance NPC to OPC commitment *in vitro* or *in vivo* (Figures 2J, 6E-F), and does not lead to altered precursor cell migration into the white matter tracts *in vivo* (Figures 5L, 5O and 6C). Therefore, our data do not support the role for exogenous HDGF in OPC specification from NPCs, or NPC/OPC migration. Pro-proliferative properties of HDGF on PDGFRα^+^ OPCs *in vitro* and *in vivo* (Figures 2M, 5Q) are supported by previous literature, where HDGF was shown to act as a mitogen for hepatocytes and muscle cells (Everett *et al*., 2001; Enomoto *et al*., 2002). Interestingly, high expression of HDGF is observed in a variety of cancers, such gliomas, bladder and ovarian cancers as well as hepatocellular carcinomas (Song *et al*., 2014; Enomoto *et al*., 2015; Giri *et al*., 2016; Yang *et al*., 2017b; Zhang *et al*., 2019). HDGF knockdown in glioma cells, which originate from NPCs and/or OPCs (Ilkhanizadeh *et al*., 2014), inhibits their proliferation via inhibition of PI3K/Akt signalling pathway (Song *et al*., 2014). Our results support and extend this report by showing exogenous HDGF can increase proliferation of “normal” PDGFRα^+^ OPCs *in vitro* and *in vivo*. Whether this occurs by enhancing PI3K/Akt or modulation of any other signalling pathways remains to be determined. Notably, while exogenous HDGF increased SVZ NPC proliferation *in vitro* (Figure 2B,E-F), this effect was not recapitulated *in vivo* (Figure 4-5). As we only analyzed *in vivo* NPC proliferation on 1 and 7 days of infusion, it is possible we missed a time point at which HDGF may modulate NPC proliferation. It is also possible that a modest effect of HDGF on *in vitro* NPC proliferation is not conserved *in vivo*, or that a higher concentration of HDGF is required to increase SVZ NPC proliferation *in vivo*. In support of the latter, HDGF was shown to increase hepatocyte proliferation at 50 ng/ml (Enomoto *et al*., 2002), which is a 5 times higher concentration compared to what we used (10 ng/day). In addition, it is possible that endogenous HDGF may be present in the SVZ niche, and a higher amount of exogenous HDGF is needed to overcome any effects that endogenous HDGF may exert. A limitation in our 7-day infusion proliferation analysis is that we assessed the proliferation of total Sox2^+^ and PDGFRα^+^ cells, which can represent a mixture of SVZ-derived and resident parenchymal progenitors (Figure 5). It is possible that if we would have increased our sample size and lineage traced SVZ cells with YFP expression, we would have been able to determine additional differences in the SVZ lineage traced cells. Finally, it is possible that HDGF exerts NPC pro-proliferative effects only in the presence of EGF and/or FGF, which are present in the NPC monolayer media (Figure 2), and may not be present in similar concentrations *in vivo*.

It is well established that neuronal activity and neuronally secreted ligands modulate OPC density, proliferation and differentiation in homeostatic, injury or disease conditions (Gibson *et al*., 2014; Orduz *et al*., 2015; Venkatesh *et al*., 2015; Voronova *et al*., 2017; Ortiz *et al*., 2019). For example, neuronally secreted Neuroligin-3 promotes OPC proliferation, whereas interneuron-secreted fractalkine (CX3CL1) promotes OPC differentiation (Venkatesh *et al*., 2015; Voronova *et al*., 2017; Watson *et al*., 2021). HDGF is expressed and secreted by neurons (Zhou *et al*., 2004); however, whether neuronally secreted HDGF is responsible for neuron-specific effects on OPCs remains to be addressed. Interestingly, another HDGF family member, HDGF-related protein 3 (HRP3), is enriched in CNS and peripheral nervous system (PNS) white matter tracts during myelination and remyelination (Kerman *et al*., 2020). From the two isoforms of HRP-3, only the overexpression of the second isoform HRP3-II in PNS neurons increases Schwann cell numbers and myelination in neuron-glia co-cultures (Kerman *et al*., 2020). Our results showing HDGF exerts pro-proliferative and pro-differentiation effects on SVZ precursor cells support and extend this report. Whether HDGF has an effect on Schwann cell biology in the PNS, and/or increases CNS or PNS myelination remains to be addressed.

HDGF acts on a variety of cell types and exerts anti-apoptotic, pro-angiogenic, and neurotrophic effects (Everett et al., 2001; Enomoto et al., 2002; Zhou et al., 2004; Enomoto et al., 2015). Notably, other neuronal ligands act as pleiotropic molecules on a variety of cell types. For example, Neuregulin-1 (NRG1) acts on Schwann cells, oligodendrocytes, motoneurons, macrophages, Th1 cells and microglia to regulate myelination, neuroprotection and inflammation (Alizadeh *et al*., 2018; Kataria *et al*., 2021 and reviewed in Mei and Xiong, 2008; Kataria *et al*., 2019). Another neuronally expressed ligand, pleiotrophin (PTN), acts as a mitogen as well as neurotrophic and immunomodulatory molecule (Li *et al*., 1990; Silos-Santiago *et al*., 1996 and reviewed in González-Castillo *et al*., 2015; Sorrelle *et al*., 2017). Therefore, it is possible that HDGF has the potential to act as a pleiotropic ligand on various cells in the organism, including SVZ precursor cells.

Our results indicate that HDGF does not modulate SVZ precursor cell fates via NCL, the only receptor that has been identified to interact with and mediate the uptake of HDGF in hepatoma cells (Chen *et al*., 2015). Whether HDGF interacts with NCL in other CNS cells is currently not known. A recent large-scale mouse tissue proteomic screen identified 26 interactors of HDGF in the adult brain, however, NCL was not on this list (Skinnider *et al*., 2021). Thus, it is possible HDGF interacts with a different receptor in SVZ precursors or CNS cells at large. In the future, it will be important to determine what receptor interacts with HDGF in SVZ NPCs and/or OPCs.

In summary, we demonstrate a novel role for HDGF in the brain, where exogenous HDGF modulates SVZ NPC and OPC fates during oligodendrocyte genesis. As pro-oligodendrogenic treatments have been prioritized in clinical mouse models of neurodevelopmental and neurodegenerative disorders, our studies warrant further investigation into whether HDGF may represent a novel candidate molecule for SVZ NPC and/or OPC engagement in the diseased or injured brain.[Table table1-17590914221086340]

**Table 1. table1-17590914221086340:** Summary of Sample Sizes and Biological Replicates for Each Experiment.

Experiment	Panels	Number of Replicates	Age of mice & Strain
NPC 5DIV Differentiation *In vitro*	1D-G	3-5 biological replicates	P7, CD1
	S1	4 biological replicates	
NPC 7DIV 2° Neurospheres *In vitro*	2B	3 Biological replicates 9 Technical replicates	P7, CD1
NPC 1DIV Proliferation *In vitro*	2D-F	5 biological replicates	P7, CD1
	2G	4 biological replicates	
NPC 3DIV Proliferation *In vitro*	2I-M	3 biological replicates	P7, CD1
	2N	4 biological replicates	
	2O	3 biological replicates	
OPC 2 + 3DIV Differentiation *In vitro*	3C-D	5 biological replicates	P7, CD1
	3E	4 biological replicates	
	7E	3 biological replicates	
24hr Injection NPC Proliferation *In vivo*	4C-H	3 animals/condition from two independent litters	3-month old, NestinCre^ERT2^; RosaYFP^STOP^
7 Day Infusion NPC Proliferation *In vivo*	5C-J	3-4 animals/condition from two independent litters	3-month old, NestinCre^ERT2^; RosaYFP^STOP^
7 Day Infusion OPC Proliferation *In vivo*	5L-Q	3 animals/condition from two independent litters	3-month old, NestinCre^ERT2^; RosaYFP^STOP^
7 Day Infusion Differentiation *In vivo*	6C 6E-F 6H-I	3-4 animals/condition from two independent litters	3-month old, NestinCre^ERT2^; RosaYFP^STOP^

## Supplemental Material

sj-docx-1-asn-10.1177_17590914221086340 - Supplemental material for Hepatoma Derived Growth Factor Enhances Oligodendrocyte Genesis from Subventricular Zone Precursor Cells Click here for additional data file. Supplemental material, sj-docx-1-asn-10.1177_17590914221086340 for Hepatoma Derived Growth Factor Enhances Oligodendrocyte Genesis from Subventricular Zone Precursor Cells by Yutong Li, Nicole Leanne Dittmann and Adrianne Eve, Scovil Watson, Monique Marylin Alves de Almeida, Tim Footz, Anastassia Voronova in ASN Neuro

## Abbreviations

BCAS1: breast carcinoma amplified sequence 1
BrdU: Bromodeoxyuridine
BSA: Bovine Serum Albumin
CC: corpus callosum
CNS: central nervous system
DIV: days in vitro
EGF: epidermal growth factor
FGF: fibroblast growth factor
GFAP: Glial Fibrillary Acidic Protein
HDGF: Hepatoma Derived Growth Factor
ICV: intracerebral ventricular
LV: lateral ventricle
MBP: myelin basic protein
MDK: midkine
NCL: nucleolin-1
NPC: neural precursor cell
NRG1: Neuregulin-1
OPC: oligodendrocyte precursor cell
PDGF-AA: platelet derived growth factor AA
PDGFRα: Platelet Derived Growth Factor Receptor Alpha
PFA: paraformaldehyde
PLP: proteolipid protein
PNS: peripheral nervous system
PTN: pleiotrophin
Sox2: sex determining region Y-box 2
Str: striatum
SVZ: subventricular zone
T3: 3,3',5-Triiodo-L-thyronine
VC: vehicle control
WM: white matter
YFP: yellow fluorescent protein

## References

[bibr1-17590914221086340] Adams K. L. Riparini G. Banerjee P. Breur M. Bugiani M. Gallo V. (2020). Endothelin-1 signaling maintains glial progenitor proliferation in the postnatal subventricular zone. Nature Communications, 11(1), 2138. 10.1038/s41467-020-16028-8 PMC7195367 32358570

[bibr2-17590914221086340] Aguirre A. Dupree J. L. Mangin J. M. Gallo V. (2007). A functional role for EGFR signaling in myelination and remyelination. Nature Neuroscience, 10(8), 990–1002. 10.1038/nn1938 17618276

[bibr3-17590914221086340] Aguirre A. Gallo V. (2007). Reduced EGFR signaling in progenitor cells of the adult subventricular zone attenuates oligodendrogenesis after demyelination. Neuron Glia Biology, 3(3), 209–220. 10.1017/S1740925X08000082 18634612 PMC2696258

[bibr4-17590914221086340] Ahmed S. (2009). The culture of neural stem cells. Journal of Cellular Biochemistry, 106(1), 1–6. 10.1002/jcb.21972 19021147

[bibr5-17590914221086340] Alizadeh A. Santhosh K. T. Kataria H. Gounni A. S. Karimi-Abdolrezaee S. (2018). Neuregulin-1 elicits a regulatory immune response following traumatic spinal cord injury. Journal of Neuroinflammation, 15(1), 53. 10.1186/s12974-018-1093-9 29467001 PMC5822667

[bibr6-17590914221086340] Bacigaluppi M. Sferruzza G. Butti E. Ottoboni L. Martino G. (2020). Endogenous neural precursor cells in health and disease. Brain Research, 1730, 146619. 10.1016/j.brainres.2019.146619 31874148

[bibr7-17590914221086340] Bao C. Wang J. Ma W. Wang X. Cheng Y. (2014). HDGF: A novel jack-of-all-trades in cancer. Future oncology (London. England, 10(16), 2675–2685. 10.2217/fon.14.194 25236340

[bibr8-17590914221086340] Barak B. Zhang Z. Liu Y. Nir A. Trangle S. S. Ennis M. Levandowski K. M. Wang D. Quast K. Boulting G. L. Li Y. Bayarsaihan D. He Z. Feng G. (2019). Neuronal deletion of Gtf2i, associated with williams syndrome, causes behavioral and myelin alterations rescuable by a remyelinating drug. Nature Neuroscience, 22(5), 700–708. 10.1038/s41593-019-0380-9 31011227

[bibr9-17590914221086340] Bhat N. R. Sarlieve L. L. Rao G. S. Pieringer R. A. (1979). Investigations on myelination in vitro. Regulation by thyroid hormone in cultures of dissociated brain cells from embryonic mice. Journal of Biological Chemistry, 254(19), 9342–9344. 10.1016/S0021-9258(19)83519-6 489534

[bibr10-17590914221086340] Borrett M. J. Innes B. T. Jeong D. Tahmasian N. Storer M. A. Bader G. D. Kaplan D. R. Miller F. D. (2020). Single-Cell profiling shows murine forebrain neural stem cells reacquire a developmental state when activated for adult neurogenesis. Cell Reports, 32(6), 108022. 10.1016/j.celrep.2020.108022 32783944

[bibr11-17590914221086340] Brousse B. Magalon K. Durbec P. Cayre M. (2015). Region and dynamic specificities of adult neural stem cells and oligodendrocyte precursors in myelin regeneration in the mouse brain. Biology Open, 4(8), 980–992. 10.1242/bio.012773 26142314 PMC4542288

[bibr12-17590914221086340] Brousse B. Mercier O. Magalon K. Daian F. Durbec P. Cayre M. (2021). Endogenous neural stem cells modulate microglia and protect against demyelination. Stem Cell Reports, 16(7), 1792–1804. 10.1016/j.stemcr.2021.05.002 34087164 PMC8282429

[bibr13-17590914221086340] Butovsky O. Ziv Y. Schwartz A. Landa G. Talpalar A. E. Pluchino S. Martino G. Schwartz M. (2006). Microglia activated by IL-4 or IFN-gamma differentially induce neurogenesis and oligodendrogenesis from adult stem/progenitor cells. Molecular and Cellular Neuroscience, 31(1), 149–160. 10.1016/j.mcn.2005.10.006 16297637

[bibr14-17590914221086340] Bylund M. Andersson E. Novitch B. G. Muhr J. (2003). Vertebrate neurogenesis is counteracted by Sox1-3 activity. Nature Neuroscience, 6(11), 1162–1168. 10.1038/nn1131 14517545

[bibr15-17590914221086340] Capilla-Gonzalez V. Cebrian-Silla A. Guerrero-Cazares H. Garcia-Verdugo J. M. Quiñones-Hinojosa A. (2013). The generation of oligodendroglial cells is preserved in the rostral migratory stream during aging. Frontiers in Cellular Neuroscience, 7, 147. 10.3389/fncel.2013.00147 24062640 PMC3775451

[bibr16-17590914221086340] Cayre M. Bancila M. Virard I. Borges A. Durbec P. (2006). Migrating and myelinating potential of subventricular zone neural progenitor cells in white matter tracts of the adult rodent brain. Molecular and Cellular Neuroscience, 31(4), 748–758. 10.1016/j.mcn.2006.01.004 16481195

[bibr17-17590914221086340] Cebrian Silla A. Nascimento M. A. Redmond S. A. Mansky B. Wu D. Obernier K. Romero Rodriguez R. Gonzalez Granero S. García-Verdugo J. M. Lim D. Álvarez-Buylla A. (2021). Single-cell analysis of the ventricular-subventricular zone reveals signatures of dorsal & ventral adult neurogenesis. Elife, 10: e67436. 10.7554/eLife.67436 34259628 PMC8443251

[bibr18-17590914221086340] Chaker Z. Codega P. Doetsch F. (2016). A mosaic world: Puzzles revealed by adult neural stem cell heterogeneity. Wiley Interdisciplinary Reviews. Developmental Biology, 5(6), 640–658. 10.1002/wdev.248 27647730 PMC5113677

[bibr19-17590914221086340] Chen J. F. Liu K. Hu B. Li R. R. Xin W. Chen H. Wang F. Chen L. Li R. X. Ren S. Y. Xiao L. Chan J. R. Mei F. (2021). Enhancing myelin renewal reverses cognitive dysfunction in a murine model of Alzheimer's disease. Neuron, 109(14), 2292–2307.e2295. 10.1016/j.neuron.2021.05.012 34102111 PMC8298291

[bibr20-17590914221086340] Chen S. C. Hu T. H. Huang C. C. Kung M. L. Chu T. H. Yi L. N. Huang S. T. Chan H. H. Chuang J. H. Liu L. F. Wu H. C. Wu D. C. Chang M. C. Tai M. H. (2015). Hepatoma-derived growth factor/nucleolin axis as a novel oncogenic pathway in liver carcinogenesis. Oncotarget, 6(18), 16253–16270. 10.18632/oncotarget.3608 25938538 PMC4599268

[bibr21-17590914221086340] Coles-Takabe B. L. Brain I. Purpura K. A. Karpowicz P. Zandstra P. W. Morshead C. M. van der Kooy D. (2008). Don't look: Growing clonal versus nonclonal neural stem cell colonies. Stem Cells (Dayton, Ohio), 26(11), 2938–2944. 10.1634/stemcells.2008-0558 18757294

[bibr22-17590914221086340] Crossin K. L. Tai M. H. Krushel L. A. Mauro V. P. Edelman G. M. (1997). Glucocorticoid receptor pathways are involved in the inhibition of astrocyte proliferation. Proceedings of the National Academy of Sciences of the United States of America, 94(6), 2687–2692. 10.1073/pnas.94.6.2687 9122257 PMC20150

[bibr23-17590914221086340] Delgado A. C. Maldonado-Soto A. R. Silva-Vargas V. Mizrak D. von Känel T. Tan K. R. Paul A. Madar A. Cuervo H. Kitajewski J. Lin C. S. Doetsch F. (2021). Release of stem cells from quiescence reveals gliogenic domains in the adult mouse brain. Science (New York, N.Y.), 372(6547), 1205–1209. 10.1126/science.abg8467 34112692

[bibr24-17590914221086340] Đặng T. C. Ishii Y. Nguyen V. Yamamoto S. Hamashima T. Okuno N. Nguyen Q. L. Sang Y. Ohkawa N. Saitoh Y. Shehata M. Takakura N. Fujimori T. Inokuchi K. Mori H. Andrae J. Betsholtz C. Sasahara M. (2019). Powerful homeostatic control of oligodendroglial lineage by PDGFRα in adult brain. Cell Reports, 27(4), 1073–1089.e1075. 10.1016/j.celrep.2019.03.084 31018125

[bibr25-17590914221086340] El-Tahir H. M. Dietz F. Dringen R. Schwabe K. Strenge K. Kelm S. Abouzied M. M. Gieselmann V. Franken S. (2006). Expression of hepatoma-derived growth factor family members in the adult central nervous system. BMC Neuroscience, 7(1), 6. 10.1186/1471-2202-7-6 16430771 PMC1363353

[bibr26-17590914221086340] Enomoto H. Nakamura H. Liu W. Nishiguchi S. (2015). Hepatoma-Derived growth factor: Its possible involvement in the progression of hepatocellular carcinoma. International Journal of Molecular Sciences, 16(6), 14086–14097. 10.3390/ijms160614086 26101867 PMC4490540

[bibr27-17590914221086340] Enomoto H. Yoshida K. Kishima Y. Kinoshita T. Yamamoto M. Everett A. D. Miyajima A. Nakamura H. (2002). Hepatoma-derived growth factor is highly expressed in developing liver and promotes fetal hepatocyte proliferation. Hepatology (Baltimore. Md Medical Newsmagazine, 36(6), 1519–1527. 10.1053/jhep.2002.36935 12447878

[bibr28-17590914221086340] Everett A. D. Stoops T. McNamara C. A. (2001). Nuclear targeting is required for hepatoma-derived growth factor-stimulated mitogenesis in vascular smooth muscle cells. Journal of Biological Chemistry, 276(40), 37564–37568. 10.1074/jbc.M105109200 11481329

[bibr29-17590914221086340] Fard M. K. van der Meer F. Sánchez P. Cantuti-Castelvetri L. Mandad S. Jäkel S. Fornasiero E. F. Schmitt S. Ehrlich M. Starost L. Kuhlmann T. Sergiou C. Schultz V. Wrzos C. Brück W. Urlaub H. Dimou L. Stadelmann C. Simons M ,.… (2017). BCAS1 Expression defines a population of early myelinating oligodendrocytes in multiple sclerosis lesions. Science Translational Medicine, 9(419):eaam7816. 10.1126/scitranslmed.aam7816. 29212715 PMC7116798

[bibr30-17590914221086340] Gadea A. Aguirre A. Haydar T. F. Gallo V. (2009). Endothelin-1 regulates oligodendrocyte development. Journal of Neuroscience, 29(32), 10047–10062. 10.1523/JNEUROSCI.0822-09.2009 19675238 PMC2754292

[bibr31-17590914221086340] Gallitzendoerfer R. Abouzied M. M. Hartmann D. Dobrowolski R. Gieselmann V. Franken S. (2008). Hepatoma-derived growth factor (HDGF) is dispensable for normal mouse development. Developmental Dynamics, 237(7), 1875–1885. 10.1002/dvdy.21589 18570251

[bibr32-17590914221086340] Gibson E. M. Purger D. Mount C. W. Goldstein A. K. Lin G. L. Wood L. S. Inema I. Miller S. E. Bieri G. Zuchero J. B. Barres B. A. Woo P. J. Vogel H. Monje M. (2014). Neuronal activity promotes oligodendrogenesis and adaptive myelination in the mammalian brain. Science (New York, N.Y.), 344(6183), 1252304. 10.1126/science.1252304 PMC4096908 24727982

[bibr33-17590914221086340] Giri K. Pabelick C. M. Mukherjee P. Prakash Y. S. (2016). Hepatoma derived growth factor (HDGF) dynamics in ovarian cancer cells. Apoptosis : an international journal on programmed cell death, 21(3), 329–339. 10.1007/s10495-015-1200-7 26612514

[bibr34-17590914221086340] Goldman J. E. (1995). Lineage, migration, and fate determination of postnatal subventricular zone cells in the mammalian CNS. Journal of Neuro-Oncology, 24(1), 61–64. 10.1007/BF01052660 8523077

[bibr35-17590914221086340] Gonzalez-Perez O. Alvarez-Buylla A. (2011). Oligodendrogenesis in the subventricular zone and the role of epidermal growth factor. Brain Research Reviews, 67(1-2), 147–156. 10.1016/j.brainresrev.2011.01.001 21236296 PMC3109119

[bibr36-17590914221086340] Gonzalez-Perez O. Quiñones-Hinojosa A. (2010). Dose-dependent effect of EGF on migration and differentiation of adult subventricular zone astrocytes. Glia (8), 58, 975–983. 10.1002/glia.20979 20187143 PMC2915565

[bibr37-17590914221086340] González-Castillo C. Ortuño-Sahagún D. Guzmán-Brambila C. Pallàs M. Rojas-Mayorquín A. E. (2015). Pleiotrophin as a central nervous system neuromodulator, evidences from the hippocampus. Frontiers in Cellular Neuroscience, 8(8), 443. 10.3389/fncel.2014.00443 25620911 PMC4287103

[bibr38-17590914221086340] Graham V. Khudyakov J. Ellis P. Pevny L. (2003). SOX2 Functions to maintain neural progenitor identity. Neuron, 39(5), 749–765. 10.1016/S0896-6273(03)00497-5 12948443

[bibr39-17590914221086340] Ilkhanizadeh S. Lau J. Huang M. Foster D. J. Wong R. Frantz A. Wang S. Weiss W. A. Persson A. I. (2014). Glial progenitors as targets for transformation in glioma. Advances in Cancer Research, 121, 1–65. 10.1016/B978-0-12-800249-0.00001-9 24889528 PMC4270964

[bibr40-17590914221086340] Imayoshi I. Ohtsuka T. Metzger D. Chambon P. Kageyama R. (2006). Temporal regulation of Cre recombinase activity in neural stem cells. Genesis (New York, N.Y.: 2000), 44(5), 233–238. 10.1002/dvg.20212 16652364

[bibr41-17590914221086340] Ishimoto T. Ninomiya K. Inoue R. Koike M. Uchiyama Y. Mori H. (2017). Mice lacking BCAS1, a novel myelin-associated protein, display hypomyelination, schizophrenia-like abnormal behaviors, and upregulation of inflammatory genes in the brain. Glia, 65(5), 727–739. 10.1002/glia.23129 28230289

[bibr42-17590914221086340] Jablonska B. Aguirre A. Raymond M. Szabo G. Kitabatake Y. Sailor K. A. Ming G. L. Song H. Gallo V. (2010). Chordin-induced lineage plasticity of adult SVZ neuroblasts after demyelination. Nature Neuroscience, 13(5), 541–550. 10.1038/nn.2536 20418875 PMC4059417

[bibr43-17590914221086340] Kang S. H. Fukaya M. Yang J. K. Rothstein J. D. Bergles D. E. (2010). NG2 + CNS Glial progenitors remain committed to the oligodendrocyte lineage in postnatal life and following neurodegeneration. Neuron, 68(4), 668–681. 10.1016/j.neuron.2010.09.009 21092857 PMC2989827

[bibr44-17590914221086340] Kataria H. Alizadeh A. Karimi-Abdolrezaee S. (2019). Neuregulin-1/ErbB network: An emerging modulator of nervous system injury and repair. Progress in Neurobiology, 180, 101643. 10.1016/j.pneurobio.2019.101643 31229498

[bibr45-17590914221086340] Kataria H. Hart C. G. Alizadeh A. Cossoy M. Kaushik D. K. Bernstein C. N. Marrie R. A. Yong V. W. Karimi-Abdolrezaee S. (2021). Neuregulin-1 beta 1 is implicated in pathogenesis of multiple sclerosis. Brain : a journal of neurology, 144(1), 162–185. 10.1093/brain/awaa385 33313801 PMC7880664

[bibr46-17590914221086340] Kerman B. E. Genoud S. Kurt Vatandaslar B. Denli A. M. Georges Ghosh S. Xu X. Yeo G. W. Aimone J. B. Gage F. H. (2020). Motoneuron expression profiling identifies an association between an axonal splice variant of HDGF-related protein 3 and peripheral myelination. Journal of Biological Chemistry, 295(34), 12233–12246. 10.1074/jbc.RA120.014329 PMC7443494 32647008

[bibr47-17590914221086340] Kessaris N. Fogarty M. Iannarelli P. Grist M. Wegner M. Richardson W. D. (2006). Competing waves of oligodendrocytes in the forebrain and postnatal elimination of an embryonic lineage. Nature Neuroscience, 9(2), 173–179. 10.1038/nn1620 16388308 PMC6328015

[bibr48-17590914221086340] Lakshman N. Bourget C. Siu R. Bamm V. V. Xu W. Harauz G. Morshead C. M. (2021). Niche-dependent inhibition of neural stem cell proliferation and oligodendrogenesis is mediated by the presence of myelin basic protein. Stem Cells (Dayton, Ohio), 39(6), 776–786. 10.1002/stem.3344 PMC8248327 33529418

[bibr49-17590914221086340] Levison S. W. Goldman J. E. (1993). Both oligodendrocytes and astrocytes develop from progenitors in the subventricular zone of postnatal rat forebrain. Neuron, 10(2), 201–212. 10.1016/0896-6273(93)90311-E 8439409

[bibr50-17590914221086340] Li Y. S. Milner P. G. Chauhan A. K. Watson M. A. Hoffman R. M. Kodner C. M. Milbrandt J. Deuel T. F. (1990). Cloning and expression of a developmentally regulated protein that induces mitogenic and neurite outgrowth activity. Science (New York, N.Y.), 250(4988), 1690–1694. 10.1126/science.2270483 2270483

[bibr51-17590914221086340] Lin Y. W. Huang S. T. Wu J. C. Chu T. H. Huang S. C. Lee C. C. Tai M. H. (2019). Novel HDGF/HIF-1α/VEGF axis in oral cancer impacts disease prognosis. BMC cancer, 19(1), 1083. 10.1186/s12885-019-6229-5 31711427 PMC6849302

[bibr52-17590914221086340] Loulier K. Ruat M. Traiffort E. (2006). Increase of proliferating oligodendroglial progenitors in the adult mouse brain upon sonic hedgehog delivery in the lateral ventricle. Journal of Neurochemistry, 98(2), 530–542. 10.1111/j.1471-4159.2006.03896.x 16805844

[bibr53-17590914221086340] Maki T. Liang A. C. Miyamoto N. Lo E. H. Arai K. (2013). Mechanisms of oligodendrocyte regeneration from ventricular-subventricular zone-derived progenitor cells in white matter diseases. Frontiers in Cellular Neuroscience, 7, 275–275. 10.3389/fncel.2013.00275 24421755 PMC3872787

[bibr54-17590914221086340] Mandelkow R. Gumbel D. Ahrend H. Kaul A. Zimmermann U. Burchardt M. Stope M. B. (2017). Detection and quantification of nuclear morphology changes in apoptotic cells by fluorescence microscopy and subsequent analysis of visualized fluorescent signals. Anticancer Research, 37(5), 2239–2244. 10.21873/anticanres.11560 28476788

[bibr55-17590914221086340] Marques S. van Bruggen D. Vanichkina D. P. Floriddia E. M. Munguba H. Varemo L. Giacomello S. Falcao A. M. Meijer M. Bjorklund A. K. Hjerling-Leffler J. Taft R. J. Castelo-Branco G. (2018). Transcriptional convergence of oligodendrocyte lineage progenitors during development. Developmental Cell, 46(4), 504–517.e507. 10.1016/j.devcel.2018.07.005 30078729 PMC6104814

[bibr56-17590914221086340] Matarredona E. R. Talaverón R. Pastor A. M. (2018). Interactions between neural progenitor cells and microglia in the subventricular zone: physiological implications in the neurogenic niche and after implantation in the injured brain. Frontiers in Cellular Neuroscience, 12, 268. 10.3389/fncel.2018.00268 30177874 PMC6109750

[bibr57-17590914221086340] McKenzie I. A. Ohayon D. Li H. de Faria J. P. Emery B. Tohyama K. Richardson W. D. (2014). Motor skill learning requires active central myelination. Science (New York, N.Y.), 346(6207), 318–322. 10.1126/science.1254960 PMC6324726 25324381

[bibr58-17590914221086340] Mei L. Xiong W. C. (2008). Neuregulin 1 in neural development, synaptic plasticity and schizophrenia. Nature Reviews Neuroscience, 9(6), 437–452. 10.1038/nrn2392 18478032 PMC2682371

[bibr59-17590914221086340] Menn B. Garcia-Verdugo J. M. Yaschine C. Gonzalez-Perez O. Rowitch D. Alvarez-Buylla A. (2006). Origin of oligodendrocytes in the subventricular zone of the adult brain. The Journal of Neuroscience, 26(30), 7907–7918. 10.1523/JNEUROSCI.1299-06.2006 16870736 PMC6674207

[bibr60-17590914221086340] Nait-Oumesmar B. Decker L. Lachapelle F. Avellana-Adalid V. Bachelin C. Baron-Van Evercooren A. (1999). Progenitor cells of the adult mouse subventricular zone proliferate, migrate and differentiate into oligodendrocytes after demyelination. European Journal of Neuroscience, 11(12), 4357–4366. 10.1046/j.1460-9568.1999.00873.x 10594662

[bibr61-17590914221086340] Obernier K. Alvarez-Buylla A. (2019). Neural stem cells: Origin, heterogeneity and regulation in the adult mammalian brain. Development (Cambridge, England), 146(4): dev156059. 10.1242/dev.156059 PMC6398449 30777863

[bibr62-17590914221086340] Orduz D. Maldonado P. P. Balia M. Velez-Fort M. de Sars V. Yanagawa Y. Emiliani V. Angulo M. C. (2015). Interneurons and oligodendrocyte progenitors form a structured synaptic network in the developing neocortex. Elife, 4:e06953. 10.7554/eLife.06953 PMC4432226 25902404

[bibr63-17590914221086340] Ortega F. Gascón S. Masserdotti G. Deshpande A. Simon C. Fischer J. Dimou L. Chichung Lie D. Schroeder T. Berninger B. (2013). Oligodendrogliogenic and neurogenic adult subependymal zone neural stem cells constitute distinct lineages and exhibit differential responsiveness to Wnt signalling. Nature Cell Biology, 15(6), 602–613. 10.1038/ncb2736 23644466

[bibr64-17590914221086340] Ortiz F. C. Habermacher C. Graciarena M. Houry P. Y. Nishiyama A. Nait Oumesmar B. Angulo M. C. (2019). Neuronal activity in vivo enhances functional myelin repair. JCI insight, 5(9):e123434 10.1172/jci.insight.123434. PMC6538342 30896448

[bibr65-17590914221086340] Paredes I. Vieira J. R. Shah B. Ramunno C. F. Dyckow J. Adler H. Richter M. Schermann G. Giannakouri E. Schirmer L. Augustin H. G. Ruiz de Almodóvar C. (2021). Oligodendrocyte precursor cell specification is regulated by bidirectional neural progenitor-endothelial cell crosstalk. Nature Neuroscience, 24(4), 478–488. 10.1038/s41593-020-00788-z 33510480 PMC8411877

[bibr66-17590914221086340] Paul A. Chaker Z. Doetsch F. (2017). Hypothalamic regulation of regionally distinct adult neural stem cells and neurogenesis. Science (New York, N.Y.), 356(6345), 1383–1386. 10.1126/science.aal3839 28619719

[bibr67-17590914221086340] Picard-Riera N. Decker L. Delarasse C. Goude K. Nait-Oumesmar B. Liblau R. Pham-Dinh D. Baron-Van Evercooren A. (2002). Experimental autoimmune encephalomyelitis mobilizes neural progenitors from the subventricular zone to undergo oligodendrogenesis in adult mice. Proceedings of the National Academy of Sciences of the United States of America, 99(20), 13211–13216. 10.1073/pnas.192314199 12235363 PMC130612

[bibr68-17590914221086340] Polito A. Reynolds R. (2005). NG2-expressing Cells as oligodendrocyte progenitors in the normal and demyelinated adult central nervous system. Journal of Anatomy, 207(6), 707–716. 10.1111/j.1469-7580.2005.00454.x 16367798 PMC1571577

[bibr69-17590914221086340] Remaud S. López-Juárez S. A. Bolcato-Bellemin A. L. Neuberg P. Stock F. Bonnet M. E. Ghaddab R. Clerget-Froidevaux M. S. Pierre-Simons J. Erbacher P. Demeneix B. A. Morvan-Dubois G. (2013). Inhibition of Sox2 expression in the adult neural stem cell niche In vivo by monocationic-based siRNA delivery. Molecular therapy Nucleic acids, 2(4), e89. 10.1038/mtna.2013.8 23612115 PMC3650249

[bibr70-17590914221086340] Rowitch D. H. Kriegstein A. R. (2010). Developmental genetics of vertebrate glial-cell specification. Nature, 468(7321), 214–222. 10.1038/nature09611 21068830

[bibr71-17590914221086340] Schindelin J. Arganda-Carreras I. Frise E. Kaynig V. Longair M. Pietzsch T. Preibisch S. Rueden C. Saalfeld S. Schmid B. Tinevez J.-Y. White D. J. Hartenstein V. Eliceiri K. Tomancak P. Cardona A. (2012). Fiji: An open-source platform for biological-image analysis. Nature Methods, 9(7), 676–682. 10.1038/nmeth.2019 22743772 PMC3855844

[bibr72-17590914221086340] Serwanski D. R. Rasmussen A. L. Brunquell C. B. Perkins S. S. Nishiyama A. (2018). Sequential contribution of parenchymal and neural stem cell-derived oligodendrocyte precursor cells toward remyelination. Neuroglia (Basel, Switzerland), 1(1), 91–105. 10.3390/neuroglia1010008 PMC6335037 30662979

[bibr73-17590914221086340] Silos-Santiago I. Yeh H. J. Gurrieri M. A. Guillerman R. P. Li Y. S. Wolf J. Snider W. Deuel T. F. (1996). Localization of pleiotrophin and its mRNA in subpopulations of neurons and their corresponding axonal tracts suggests important roles in neural-glial interactions during development and in maturity. Journal of Neurobiology, 31(3), 283–296. 10.1002/(SICI)1097-4695(199611)31:3<283::AID-NEU2>3.0.CO;2-6 8910787

[bibr74-17590914221086340] Silva-Vargas V. Maldonado-Soto A. R. Mizrak D. Codega P. Doetsch F. (2016). Age-Dependent niche signals from the choroid Plexus regulate adult neural stem cells. Cell Stem Cell, 19(5), 643–652. 10.1016/j.stem.2016.06.013 27452173

[bibr75-17590914221086340] Skinnider M. A. Scott N. E. Prudova A. Kerr C. H. Stoynov N. Stacey R. G. Chan Q. W. T. Rattray D. Gsponer J. Foster L. J. (2021). An atlas of protein-protein interactions across mouse tissues. Cell, 184(15), 4073–4089.e4017. 10.1016/j.cell.2021.06.003 34214469

[bibr76-17590914221086340] Sohn J. Selvaraj V. Wakayama K. Orosco L. Lee E. Crawford S. E. Guo F. Lang J. Horiuchi M. Zarbalis K. Itoh T. Deng W. Pleasure D. (2012). PEDF Is a novel oligodendrogenic morphogen acting on the adult SVZ and corpus callosum. Journal of Neuroscience, 32(35), 12152–12164. 10.1523/JNEUROSCI.0628-12.2012 22933798 PMC3457640

[bibr77-17590914221086340] Song Y. Hu Z. Long H. Peng Y. Zhang X. Que T. Zheng S. Li Z. Wang G. Yi L. Liu Z. Fang W. Qi S. (2014). A complex mechanism for HDGF-mediated cell growth, migration, invasion, and TMZ chemosensitivity in glioma. Journal of Neuro-Oncology, 119(2), 285–295. 10.1007/s11060-014-1512-4 24986090

[bibr78-17590914221086340] Sorrelle N. Dominguez A. T. A. Brekken R. A. (2017). From top to bottom: Midkine and pleiotrophin as emerging players in immune regulation. Journal of Leukocyte Biology, 102(2), 277–286. 10.1189/jlb.3MR1116-475R 28356350 PMC5505752

[bibr79-17590914221086340] Steadman P. E. Xia F. Ahmed M. Mocle A. J. Penning A. R. A. Geraghty A. C. Steenland H. W. Monje M. Josselyn S. A. Frankland P. W. (2019). Disruption of oligodendrogenesis impairs memory consolidation in adult mice. Neuron. 105(1):150-164.e6. 10.1016/j.neuron.2019.10.013 PMC7579726 31753579

[bibr80-17590914221086340] Steiner B. Kronenberg G. Jessberger S. Brandt M. D. Reuter K. Kempermann G. (2004). Differential regulation of gliogenesis in the context of adult hippocampal neurogenesis in mice. Glia, 46(1), 41–52. 10.1002/glia.10337 14999812

[bibr81-17590914221086340] Storer M. A. Gallagher D. Fatt M. P. Simonetta J. V. Kaplan D. R. Miller F. D. (2018). Interleukin-6 regulates adult neural stem cell numbers during normal and abnormal post-natal development. Stem Cell Reports, 10(5), 1464–1480. 10.1016/j.stemcr.2018.03.008 29628394 PMC5995693

[bibr82-17590914221086340] Venkatesh H. S. Johung T. B. Caretti V. Noll A. Tang Y. Nagaraja S. Gibson E. M. Mount C. W. Polepalli J. Mitra S. S. Woo P. J. Malenka R. C. Vogel H. Bredel M. Mallick P. Monje M. (2015). Neuronal activity promotes glioma growth through neuroligin-3 secretion. Cell, 161(4), 803–816. 10.1016/j.cell.2015.04.012 25913192 PMC4447122

[bibr83-17590914221086340] Voronova A. Yuzwa S. A. Wang B. S. Zahr S. Syal C. Wang J. Kaplan D. R. Miller F. D. (2017). Migrating interneurons secrete fractalkine to promote oligodendrocyte formation in the developing mammalian brain. Neuron, 94(3), 500–516.e509. 10.1016/j.neuron.2017.04.018 28472653

[bibr84-17590914221086340] Wang F. Ren S.-Y. Chen J.-F. Liu K. Li R.-X. Li Z.-F. Hu B. Niu J.-Q. Xiao L. Chan J. R. Mei F. (2020). Myelin degeneration and diminished myelin renewal contribute to age-related deficits in memory. Nature Neuroscience, 23(4), 481–486. 10.1038/s41593-020-0588-8 32042174 PMC7306053

[bibr85-17590914221086340] Wang S. Chandler-Militello D. Lu G. Roy N. S. Zielke A. Auvergne R. Stanwood N. Geschwind D. Coppola G. Nicolis S. K. Sim F. J. Goldman S. A. (2010). Prospective identification, isolation, and profiling of a telomerase-expressing subpopulation of human neural stem cells, using sox2 enhancer-directed fluorescence-activated cell sorting. Journal of Neuroscience, 30(44), 14635–14648. 10.1523/JNEUROSCI.1729-10.2010 21048121 PMC3358973

[bibr86-17590914221086340] Wanschura S. Schoenmakers E. F. Huysmans C. Bartnitzke S. Van de Ven W. J. Bullerdiek J. (1996). Mapping of the gene encoding the human hepatoma-derived growth factor (HDGF) with homology to the high-mobility group (HMG)-1 protein to Xq25. Genomics, 32(2), 298–300. 10.1006/geno.1996.0122 8833162

[bibr87-17590914221086340] Watson A. E. S. de Almeida M. M. A. Dittmann N. L. Li Y. Torabi P. Footz T. Vetere G. Galleguillos D. Sipione S. Cardona A. E. Voronova A. (2021). Fractalkine signaling regulates oligodendroglial cell genesis from SVZ precursor cells. Stem Cell Reports, 16(8), 1968–1984. 10.1016/j.stemcr.2021.06.010 34270934 PMC8365111

[bibr88-17590914221086340] Xapelli S. Agasse F. Grade S. Bernardino L. Ribeiro F. F. Schitine C. S. Heimann A. S. Ferro E. S. Sebastião A. M. De Melo Reis R. A. Malva J. O. (2014). Modulation of subventricular zone oligodendrogenesis: A role for hemopressin? Frontiers in Cellular Neuroscience, 8, 59. 10.3389/fncel.2014.00059 24578683 PMC3936357

[bibr89-17590914221086340] Xin W. Chan J. R. (2020). Myelin plasticity: Sculpting circuits in learning and memory. Nature Reviews Neuroscience, 21(12), 682–694. 10.1038/s41583-020-00379-8 33046886 PMC8018611

[bibr90-17590914221086340] Xing Y. L. Röth P. T. Stratton J. A. Chuang B. H. Danne J. Ellis S. L. Ng S. W. Kilpatrick T. J. Merson T. D. (2014). Adult neural precursor cells from the subventricular zone contribute significantly to oligodendrocyte regeneration and remyelination. Journal of Neuroscience, 34(42), 14128–14146. 10.1523/JNEUROSCI.3491-13.2014 25319708 PMC6705285

[bibr91-17590914221086340] Yang J. Cheng X. Qi J. Xie B. Zhao X. Zheng K. Zhang Z. Qiu M. (2017a). EGF Enhances oligodendrogenesis from glial progenitor cells. Frontiers in Molecular Neuroscience, 10, 106. 10.3389/fnmol.2017.00106 28442994 PMC5387051

[bibr92-17590914221086340] Yang Y. Liang S. Li Y. Gao F. Zheng L. Tian S. Yang P. Li L. (2017b). Hepatoma-derived growth factor functions as an unfavorable prognostic marker of human gliomas. Oncology Letters, 14(6), 7179–7184. 10.3892/ol.2017.7180 29344149 PMC5754909

[bibr93-17590914221086340] Yuzwa S. A. Borrett M. J. Innes B. T. Voronova A. Ketela T. Kaplan D. R. Bader G. D. Miller F. D. (2017). Developmental emergence of adult neural stem cells as revealed by single-cell transcriptional profiling. Cell Reports, 21(13), 3970–3986. 10.1016/j.celrep.2017.12.017 29281841

[bibr94-17590914221086340] Zhang C. Chang X. Chen D. Yang F. Li Z. Li D. Yu N. Yan L. Liu H. Xu Z. (2019). Downregulation of HDGF inhibits the tumorigenesis of bladder cancer cells by inactivating the PI3K-AKT signaling pathway. Cancer Management and Research, 11, 7909–7923. 10.2147/CMAR.S215341 31692549 PMC6710542

[bibr95-17590914221086340] Zhou Z. Yamamoto Y. Sugai F. Yoshida K. Kishima Y. Sumi H. Nakamura H. Sakoda S. (2004). Hepatoma-derived growth factor is a neurotrophic factor harbored in the nucleus. Journal of Biological Chemistry, 279(26), 27320–27326. 10.1074/jbc.M308650200 15140875

[bibr96-17590914221086340] Zywitza V. Misios A. Bunatyan L. Willnow T. E. Rajewsky N. (2018). Single-Cell transcriptomics characterizes cell types in the subventricular zone and uncovers molecular defects impairing adult neurogenesis. Cell Reports, 25(9), 2457–2469.e2458. 10.1016/j.celrep.2018.11.003 30485812

