# Supplementary material for: Hepatoma Derived Growth Factor Enhances Oligodendrocyte Genesis from Subventricular Zone Precursor Cells
Source: ASN Neuro. 2022 Mar 16;14:17590914221086340. doi: 10.1177/17590914221086340 (PMC8943302; doi:10.1177/17590914221086340)
Supplement: sj-docx-1-asn-10.1177_17590914221086340 - Supplemental material for Hepatoma Derived Growth Factor Enhances Oligodendrocyte Genesis from Subventricular Zone Precursor Cells [file sj-docx-1-asn-10.1177_17590914221086340.docx]

**Supplemental Information**


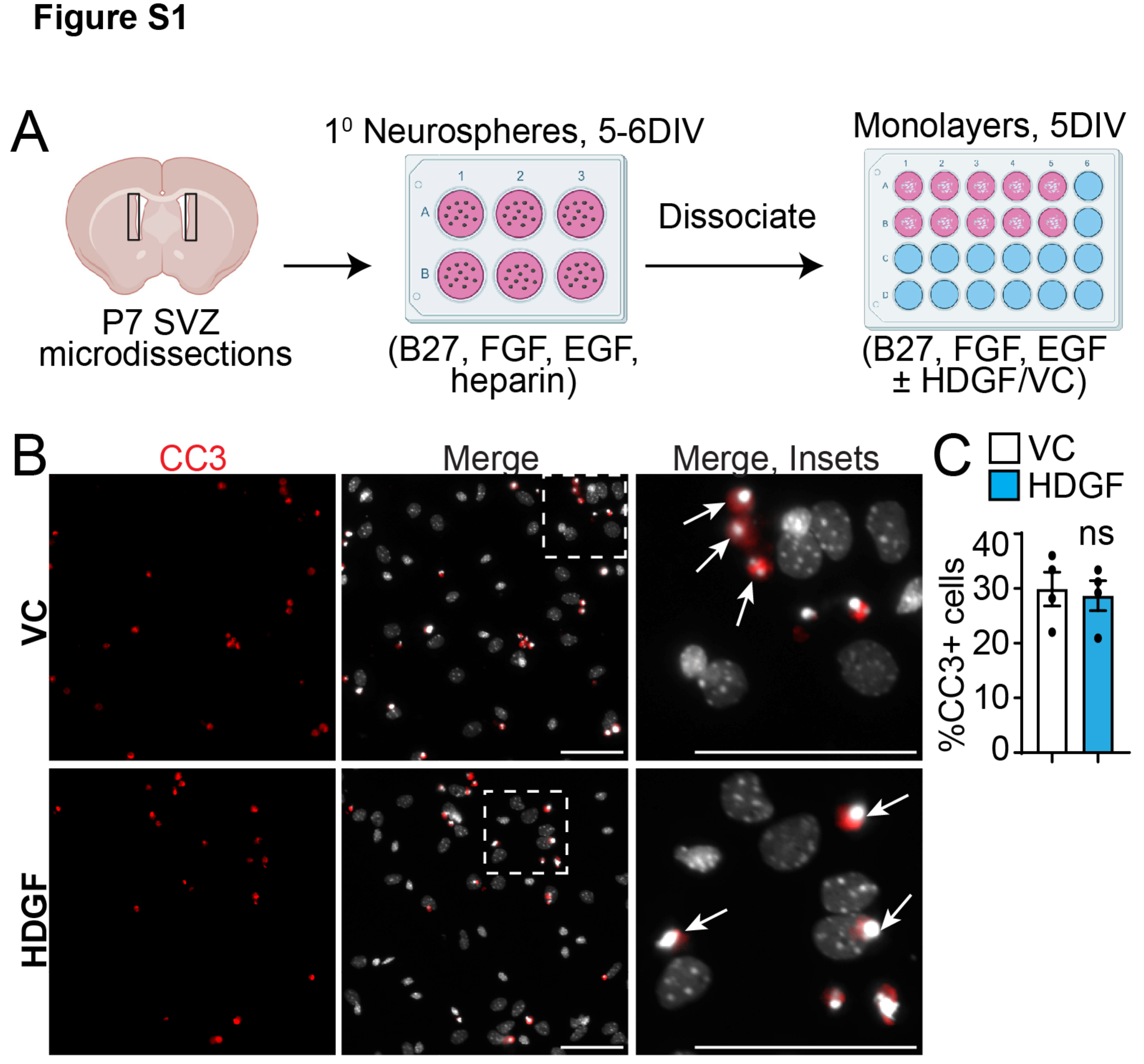


**Figure S1. HDGF does not affect apoptosis. A.** Schematic: primary neurospheres were generated from microdissected P7 SVZ, dissociated and cultured in media containing 2% B27, 10 ng/mL FGF, 20 ng/mL EGF and 2 μg/ml heparin sodium salt for 5-6 DIV followed by monolayer cultures in NPC media containing 2% B27, 10 ng/mL FGF and 20 ng/mL EGF for 5DIV with 10 ng/ml HDGF or VC**.** **B.** Representative images of NPCs cultured with VC (top) or HDGF (bottom) and immunostained for CC3 (red). Insets in “Merge” are shown in the right column. Arrows indicate CC3+ cells and contain condensed nuclei. Cells were counterstained with Hoechst 33258 (grey in merge). **C.** Quantification of **B** for the proportion of CC3+ cells in VC (white bars) or HDGF (blue bars). ns = not significant. n=4 independent experiments, at least 1000 cells per group per replicate. CC3+ cells were expressed as % of total Hoechst+ cells. Scale bars are 50 µm. Error bars represent SEM.


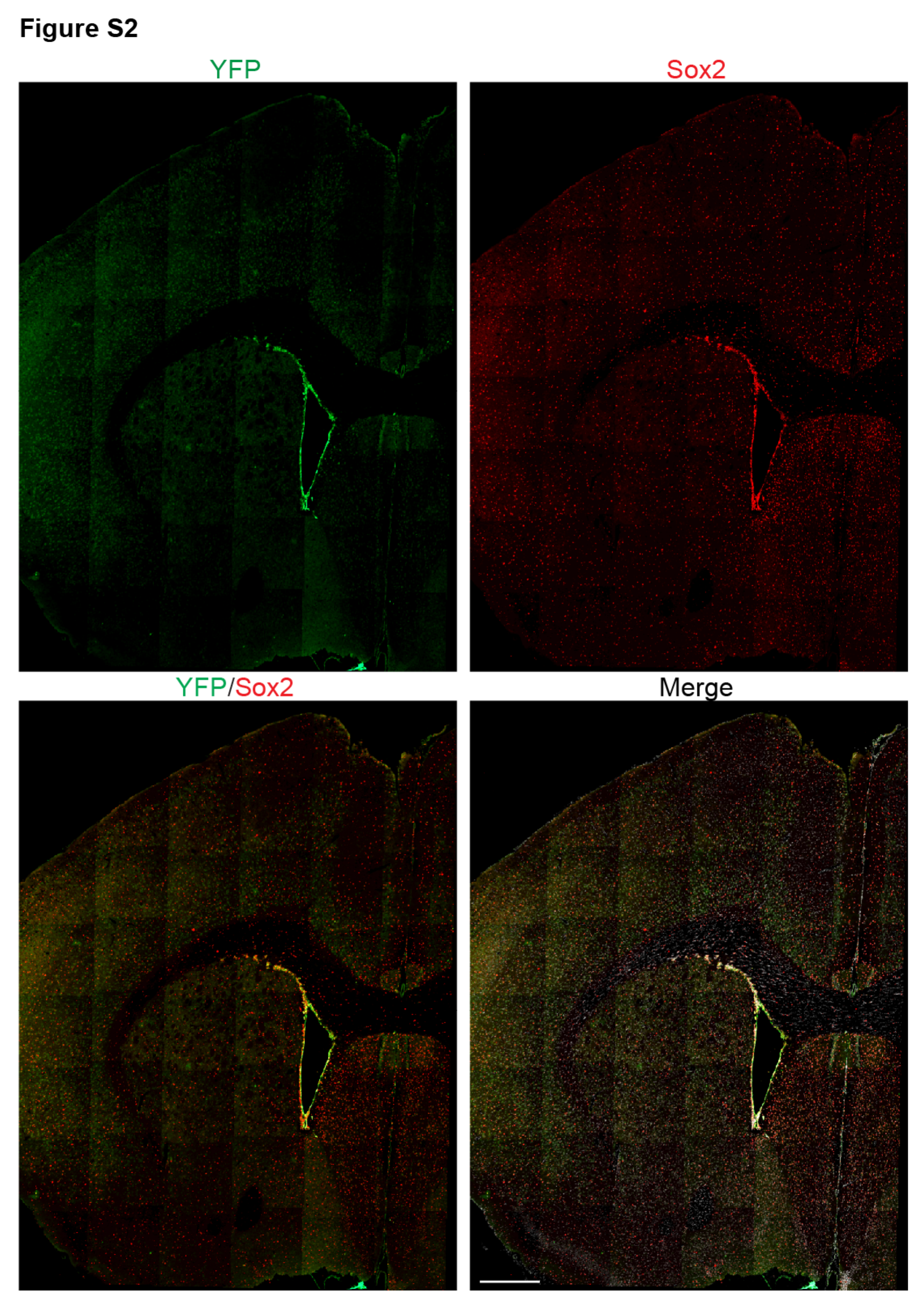


**Figure S2. YFP^+^ cells are highly enriched in the SVZ in the NestinCreERT^2^; RosaYFP^STOP^ mice.** 3-month old NestinCreERT^2^;RosaYFP^STOP^ were injected with tamoxifen for 5 days. 72h after last tamoxifen injection, mice were perfused. Brains were cryosectioned coronally and stained with anti-YFP (green) and Sox2 (red). Note enrichment and overlap of YFP and Sox2 signal in the SVZ surrounding the ventricle. Sections were counterstained with Hoechst 33258 (grey in merge). Scale bar is 500 μm.
